# NIR-II responsive PEGylated nickel nanoclusters for photothermal enhanced chemodynamic synergistic oncotherapy

**DOI:** 10.7150/thno.70841

**Published:** 2022-05-01

**Authors:** Yong Qian, Jiahui Zhang, Jinglu Zou, Xingyu Wang, Xiangfu Meng, Hongji Liu, Yefeng Lin, Qianwang Chen, Lei Sun, Wenchu Lin, Hui Wang

**Affiliations:** 1 High Magnetic Field Laboratory, Hefei Institutes of Physical Science, Chinese Academy of Sciences, Hefei 230031, Anhui, P. R. China.; 2 University of Science and Technology of China, Hefei, 230026, Anhui, P. R. China.; 3 Department of Stomatology, the Second Affiliated Hospital of Anhui Medical University, Hefei 230601, Anhui, P.R. China.; 4 The Anhui Key Laboratory of Condensed Matter Physics at Extreme Conditions, Hefei Institutes of Physical Science, Chinese Academy of Sciences, Hefei 230031, Anhui, P. R. China.; 5 Key Laboratory of High Magnetic Field and Ion Beam Physical Biology, Hefei Institutes of Physical Science, Chinese Academy of Sciences, Hefei 230031, Anhui, China.

**Keywords:** urchin-like nanostructure, nickel nanocluster, NIR-II photothermal therapy, chemodynamic therapy, synergistic effect

## Abstract

**Rationale:** All kinds of non-metal and metal-based nanozymes have been extensively explored as Fenton agents for Chemodynamic therapy (CDT). However, the low catalytic efficiency of non-metallic nanozymes and the susceptibility to oxidation and long-term toxicity of metallo-nanozymes limit their potential in CDT.

**Methods:** In this study, we report a magneto-solvothermal method to tune the crystallinity and shape of polyethylene glycol (PEG)-ylated urchin-like nickel nanoclusters (named as 9T-PUNNC) at a high magnetic field with an intensity of 9 T for enhanced combined photothermal-chemodynamic therapy.

**Results:** The needle-like protrusions on the surface of 9T-PUNNC can effectively increase the reception of NIR light in second NIR window (NIR-II) and transform it into local hyperthermia, achieving effective photothermal treatment. The light and heat generated by NIR-II further promotes the release of Ni^2+^ and improves the ability of Ni^2+^-mediated chemodynamic therapy (CDT). In addition, the surface coating of PEG on the surface of 9T-PUNNC improves its stability and biocompatibility of nanocrystals.* In vitro* and *in vivo* results indicate that the 9T-PUNNC could efficiently kill tumor cells (nearly 12 times more than control group) and inhibit tumor growth (nearly 9 times smaller than control group) under NIR-II irradiation through the synergistic effect of combined treatments.

**Conclusions:** we developed a novel synthetic strategy to tune crystallinity and shape of PUNNC for enhanced NIR-II responsive photothermal conversion efficiency and accelerated acid-induced dissolution for improved ·OH generation. Such 9T-PUNNC enable a combined chemodynamic-photothermal treatment to provide superior therapeutic efficacy due to their highly synergistic effect.

## Introduction

Chemodynamic therapy (CDT), an emerging and alternative tumor therapeutic method, has received widespread attention due to its non-invasive therapeutic modality and low side effect 1-3. The CDT based on Fenton reaction can be *in situ* realized by generating highly toxic hydroxyl radicals (·OH) within the tumor tissues, leading to cell apoptosis and tumor growth suppression 4-7. Up to now, all kinds of non-metallic and metal-based nanozymes have been widely used as Fenton agents for CDT 8-10. Although non-metallic nanozyme can catalyze Fenton reaction and produce the toxic ·OH, the low efficiency limits its clinical application 10-13. Instead, multivalent metal elements (Fe, Mn, Cu, etc.) with outstanding catalytic efficiency have been greatly potential for CDT of cancer 14-16. However, due to the characteristic of being easily oxidized and long-term toxicity, metal-based nanozymes can cause oxidative stress in non-specific delivery and affect the rate of Fenton reaction 17-19. Nickel-based nanomaterials have started to receive attention for CDT in the recent year due to their relative resistance to oxidation, and the ability to undergo Fenton-like reaction 20.

The combined treatment of photothermal-chemodynamic therapy is considered as a promising strategy to exert synergistic effect in cancer treatment 21-24, which not only increases the local temperature for hyperthermia but also accelerates Fenton reaction for enhanced CDT 25-28. Recently, the application of NIR-II irradiation further makes photothermal therapy (PTT) attractive for cancer treatment because of its advantages including deep penetration into tissues and low tissue scattering 29-32. Although much progress has been achieved in combined photothermal-chemodynamic therapy, the iron-based nanomaterials lack enough stability and NIR-II absorption, leading to the limited therapeutic efficacy through the synergistic effect 33. The excellent stability and optical absorbance of nickel nanoparticles with catalytic properties endow them with great potential for NIR-II photothermal agent to enhance chemodynamic therapy 34-36. Thus, it is very meaningful to develop biocompatible nickel-based nanomaterials for combined photothermal-chemodynamic therapy.

**Figure 1:**
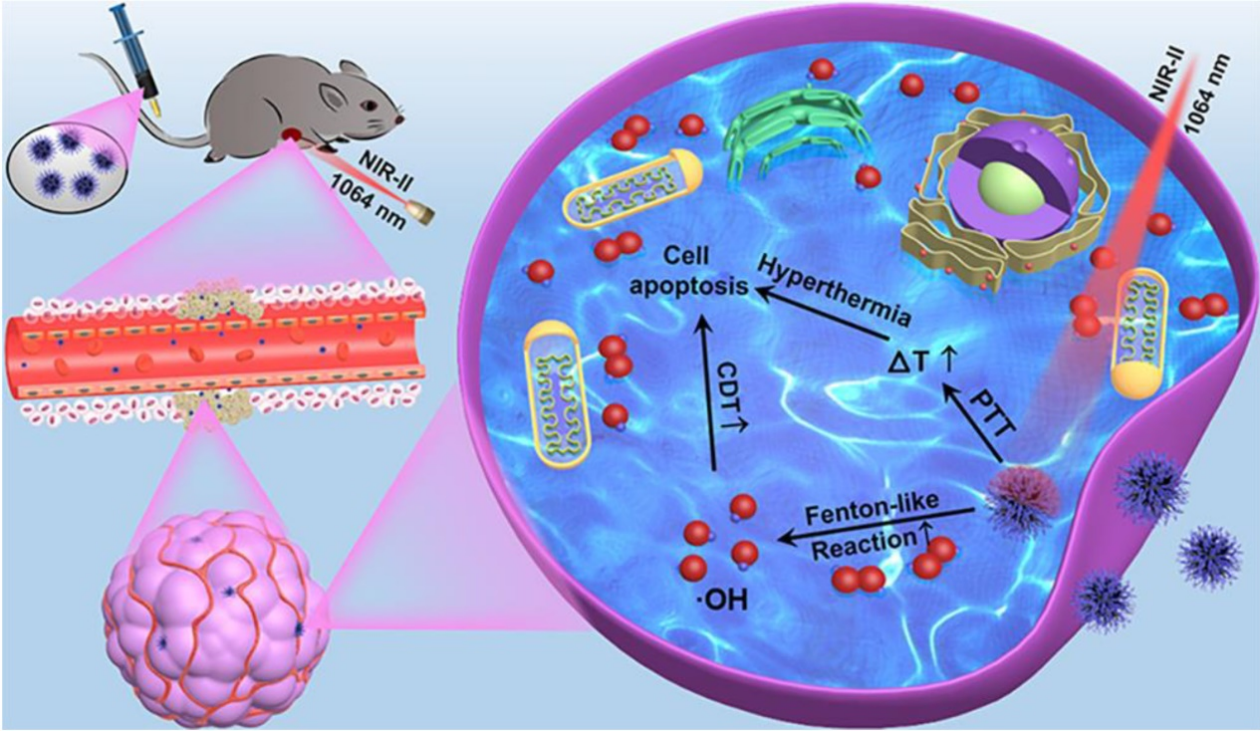
Schematic illustration of 9T-PUNNC nanoparticle-mediated photothermal enhanced chemodynamic synergistic treatment.

In this manuscript, we created a kind of PEGylation urchin-like nickel nanoclusters (PUNNCs) for combined NIR-II responsive photothermal-chemodynamic therapy (Figure 1). Such PUNNCs with prominent needle protrusions were synthesized using a magneto-solvothermal method, in which the external high magnetic field (9 T) could tune the needle length and the crystallinity of the PUNNCs 37. The ionization of 9T-PUNNCs dispersed in an acid solution can decompose hydrogen peroxide into toxic ·OH for killing cancer cells. The 9T-PUNNCs can effectively absorb and convert the NIR-II light into heat for hyperthermia treatment 38, 39. *In vivo* results indicated pronounced inhibition of tumor growth by the PUNNCs through the synergistic effect of combined chemodynamic-photothermal treatments.

## Results and Discussion

**Figure 2:**
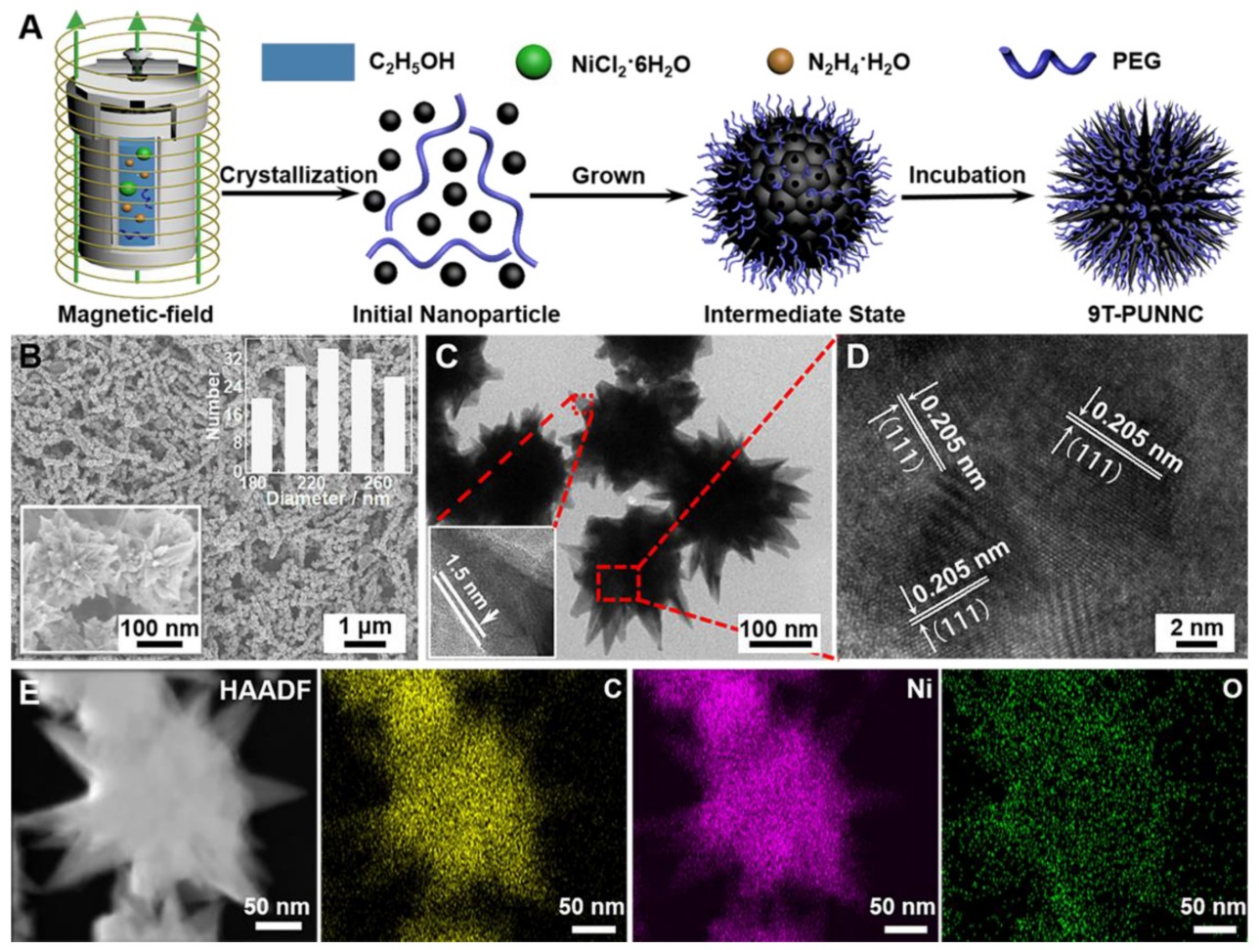
** Formation and structure of the 9T-PUNNC.** (**A**) Schematic diagram of the synthesis. (**B**) SEM images (inset, left bottom) and size distribution (inset, top right). (**C**) TEM image and high-magnification images of a single needle (inset). (**D**) HRTEM image. (**E**) HAADF and corresponding element mappings (for C, O and Ni). Scale bar, 50 nm.

The 9T-PUNNC was synthesized via a one-pot magneto-solvothermal method using nickel chloride hexahydrate and PEG as precursors under a high magnetic field of 9 T. The formation of the 9T-PUNNC should contain three steps (Figure 2A). In stage I, the Ni^2+^ dispersed in ethanol was reduced by hydrazine hydrate, resulting in the formation of nickel nanocrystals. In stage II, the Ni nanocrystals aggregated into larger secondary clusters due to their magnetic interactions and supersaturated solution and the Ni clusters were constantly covered due to the presence of PEG. In stage III, the Ni nanocrystals outside of the clusters further grew along some special crystals face induced by an external high magnetic field, resulting in the increased length of needles on their surface due to violent thermal movement and rolling of Ni nanoclusters in the reaction system. Although such magneto-solvothermal method can tune structural characteristics of functional nanomaterials, the popularity of the synthetic methods is limited by complex magnetic field equipment and chemical reaction process 40, 41.

The 9T-PUNNC has an urchin-like shape with a size distribution of 230 ± 50 nm (Figure 2B). The irregular needle protrusions with ~1.5 nm of PEG covered on the surface of the 9T-PUNNC could be observed (Figure 2C). High magnification TEM (HRTEM) image of middle position in a single 9T-PUNNC demonstrates the existence of many nanocrystals with an average size of ~10 nm, indicating the existence of cluster structure. In addition, these nanocrystals have a lattice spacing of 0.205 nm, which is consistent with the (111) plane of face-centered cubic (fcc) Ni (Figure 2D) 34. The element mappings of a single 9T-PUNNC show the existence of C, Ni and O, in which C and O elements come from PEG coating (Figure 2E). Notably, the more increase in the magnetic field, the greater degree of protrusions on the surface of the nanoclusters, which has been confirmed by the TEM images and element mappings of other PUNNCs (Supplementary Figure S1 and S2).

**Figure 3:**
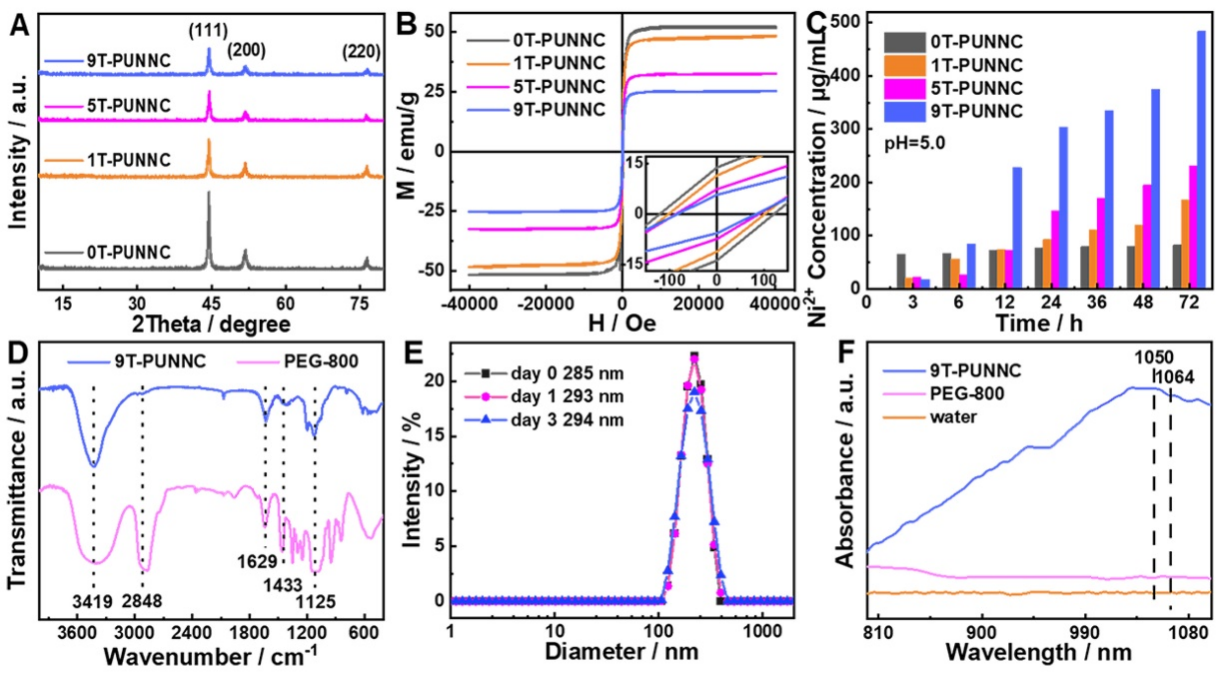
(**A, B and C**) **XRD patterns, hysteresis loops and dissolved rate of the PUNNCs synthesized under different magnetic field strengths.** The inset in b is the expanded plot measured at room temperature. (**D**) FT-IR spectra of the as-synthesized 9T-PUNNC and PEG. (**E**) Hydrodynamic sizes of the 9T-PUNNC within 3 days. (**F**) UV-vis-NIR absorption of the 9T-PUNNC, PEG and pure water.

Figure 3A shows the typical X-ray diffraction (XRD) patterns of PUNNCs synthesized under different magnetic field strengths. Three peaks at 44.68°, 52.06° and 76.48° come from the diffraction of (111), (200) and (220) crystal planes of face-centered cubic Ni (PDF 04-0850, a=3.524 Å) 42. Notably, the diffraction intensity of the corresponding crystal face in different PUNNCs was decreased with the increase of the magnetic field 43. In addition, the saturation magnetization of the PUNNCs is inversely proportional to the strength of the applied magnetic field (Figure 3B) 44. The acid-induced dissolution rate of the PUNNCs is proportional to the strength of the applied magnetic field (Figure 3C). All above results showed that the PUNNCs synthesized under a higher magnetic field demonstrated lower crystallinity, resulting from magnetic field-inducted anisotropic growth and defects formation of the PUNNCs 45.

The PUNNCs has prominent absorption peaks positions that are the same as fourier transform infrared (FT-IR) absorption of PEG (Figure 3D, Supplementary Figure S3), which confirms the presence of PEG on the surface of the 9T-PUNNC 46. The hydrophilic functional groups endow the 9T-PUNNC with negative surface charge (Supplementary Figure S4) and good dispersity in water, PBS solution, and cell culture medium containing serum (Supplementary Figure S5) 47. No noticeable change was observed in the hydrodynamic diameter of the 9T-PUNNC within 3 days and 7 days (Figure 3E, Supplementary Figure S6), indicating that the 9T-PUNNC has much better stability than that of 9T-UNNC without PEG coating in aqueous solution. Importantly, the UV-Vis-NIR absorption spectrum of the 9T-PUNNC and other PUNNCs (Figure 3F, Supplementary Figure S7) shows a broad absorption with a center of 1050 nm, which indicates their great potential as a NIR-II responsive agent for PTT.

**Figure 4:**
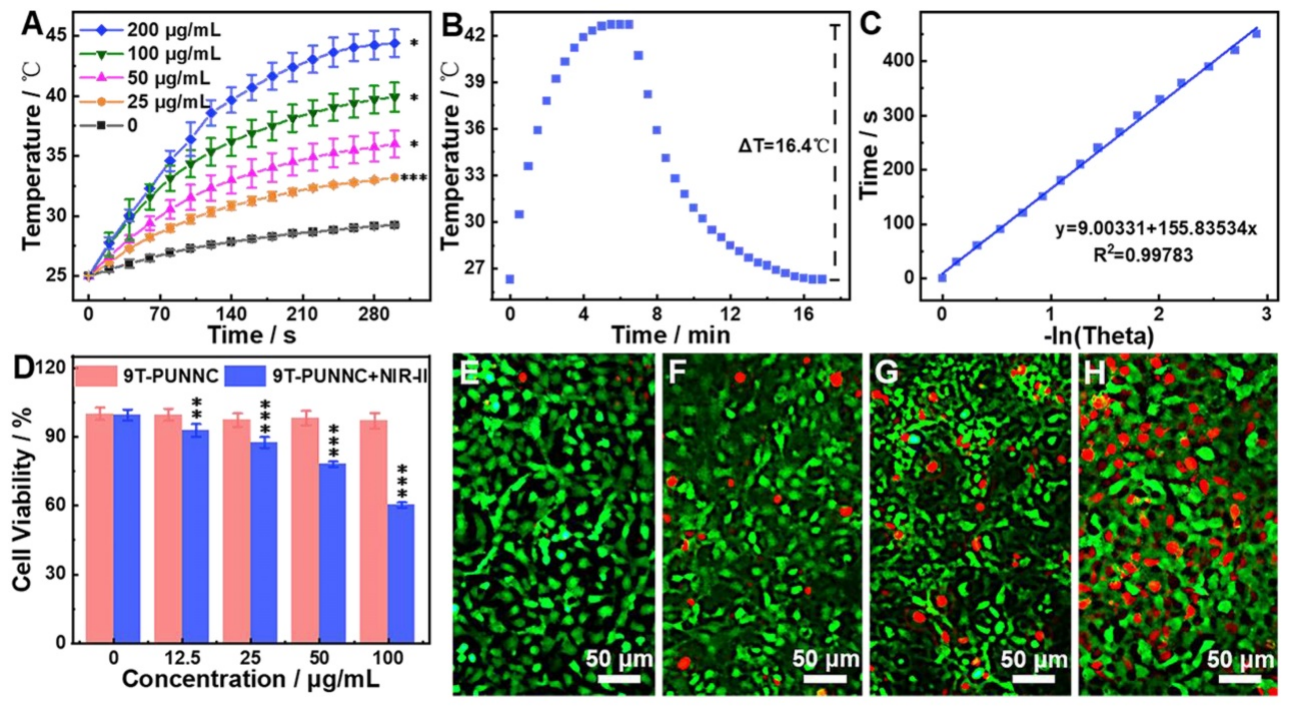
** Photothermal properties.** (**A**) Temperature change of pure water and water containing different concentrations of the 9T-PUNNC under NIR-II irradiation for 5 min. (**B**) Temperature change of the 9T-PUNNC in aqueous (200 µg mL^-1^) under NIR-II irradiation, the irradiation was turned off 390 sec after the initiation of irradiation (cooling period). (**C**) Linear time data versus *-ln θ* obtained from the cooling period shown in (B). (**D**) Cell viability of 4T1 cells treated by the 9T-PUNNC with or without NIR-II irradiation for 5 min. (**E-H**) Confocal images of 4T1 cells treated by the 9T-PUNNC at different concentrations (e, 0 µg mL^-1^; f, 25 µg mL^-1^; g, 50 µg mL^-1^; h, 100 µg mL^-1^) with a 5 min-exposure to NIR-II irradiation at pH = 7.4, and double-stained with calcein-AM/PI. Scale bar, 50 µm. The irradiation wavelength and power are 1064 nm and 0.8 W cm^-2^, respectively.

The temperature change is proportional to the concentration of the 9T-PUNNC and the temperature change of pure water is relatively low under 1064 nm laser irradiation (Figure 4A) 38. Based on the calculated heat transfer time constant (Figure 4B) and the reported photothermal conversion efficiency (*PCE*) method (Figure 4C), the *PCE* of the 9T-PUNNC was 20.93% 48. The *PCE* of the 9T-PUNNC is lower than that of the reported NIR-II photothermal agents while the absorbance wavelength of 9T-PUNNC is higher than that of the reported Ni-based photothermal agents (Supplementary Table S1 and S2). The heating of the 9T-PUNNC has no change during five cycles (Supplementary Figure S8), indicating its great photothermal stability 49. Notably, the PUNNCs synthesized under higher magnetic field strengths possess a higher *PCE* (Supplementary Figure S9 and Table S3), which may result from the increased specific surface area (Supplementary Figure S10) and an increase in the concentration of free electrons due to decreased crystallinity 50.

4T1 cell line was selected as a model to assess *in vitro* photothermal anticancer ability of the 9T-PUNNC. More than 94% of cells survived after being treated by the 9T-PUNNC (100 μg mL^-1^) for 72 h without NIR-II irradiation (Figure 4D). However, NIR-II irradiation effectively reduced cell viability to ~60% in the presence of the 9T-PUNNC at 100 μg mL^-1^. Notably, alone NIR-II irradiation has no detectable cytotoxicity, revealing an excellent photothermal effect of the 9T-PUNNC *in vitro* (Figure 4D). The confocal fluorescent images of 9T-PUNNC treated-4T1 cells co-stained by calcein-AM (green, live cells) and PI (red, dead cells) further revealed the NIR-II-induced cell death, which is proportional to the concentration of the 9T-PUNNC (Figure 4E-H).

**Figure 5:**
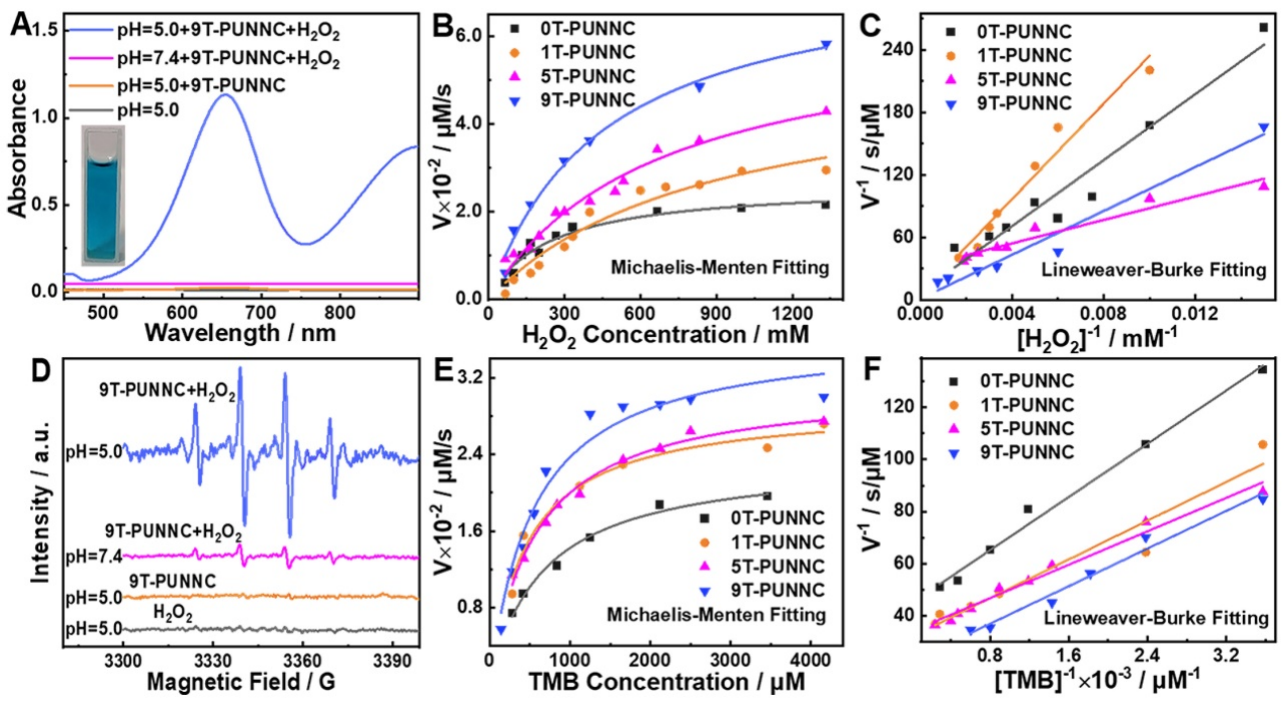
** Catalytic performance.** (**A**) UV-Vis-NIR absorbance of TMB solution under different processing conditions. (**B and C**) Michaelis-Menten fitting curves and Lineweaver-Burke fitting (double reciprocal) of ·OH generation velocities versus H_2_O_2_ in the presence of PUNNCs and TMB. (**D**) ESR signals of DMPO solution under different processing conditions. (**E and F**) Michaelis-Menten fitting curves and Lineweaver-Burke fitting (double reciprocal) of ·OH generation velocities versus TMB with the presence of PUNNCs and H_2_O_2_.

The apparent color change and absorbance at 652 nm of 3,3',5,5'-tetramethylbenzidine (TMB, ·OH scavenger) only be observed in the simultaneous presence of the 9T-PUNNC, H_2_O_2_, and acid solution (Figure 5A), which indicates that the 9T-PUNNC can decompose H_2_O_2_ into ·OH and lead to the oxidization of TMB under acidic conditions. Such catalytic activities of the PUNNCs are proportional to their pH value, concentrations, reaction times and magnetic field strength (Figure 5B-C, Supplementary Figure S11-15). The electron spin resonance (ESR) technique was utilized to prove further the catalytic activities of the PUNNCs for the generation of ·OH (Figure 5D, Supplementary Figure S16). The *K_m_* and *V_max_* of the 9T-PUNNC catalytic reaction can be determined by the Lineweaver-Burk diagram (Figure 5E-F). It is found that the PUNNCs synthesized under a higher magnetic field manifest markedly enhanced catalytic activity, which may due to the low crystallinity promoting the release of Ni^2+^ and the acceleration of the Fenton-like reaction (Supplementary Table S3) 51. The possible mechanism of Ni^2+^-mediated CDT should include two processes: one is that Ni^2+^ dissolved in an acidic environment can provide an electron for H_2_O_2_ to generate ·OH (Equation 1); the other one is that Ni^3+^ take an electron from H_2_O_2_ to produce O_2_ (Equation 2). Such electron cycle between Ni^3+^/Ni^2+^ can keep promoting the degradation of H_2_O_2_ to form ·OH and O_2_ 20.


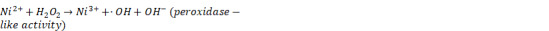

(1)


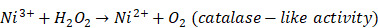

(2)

**Figure 6:**
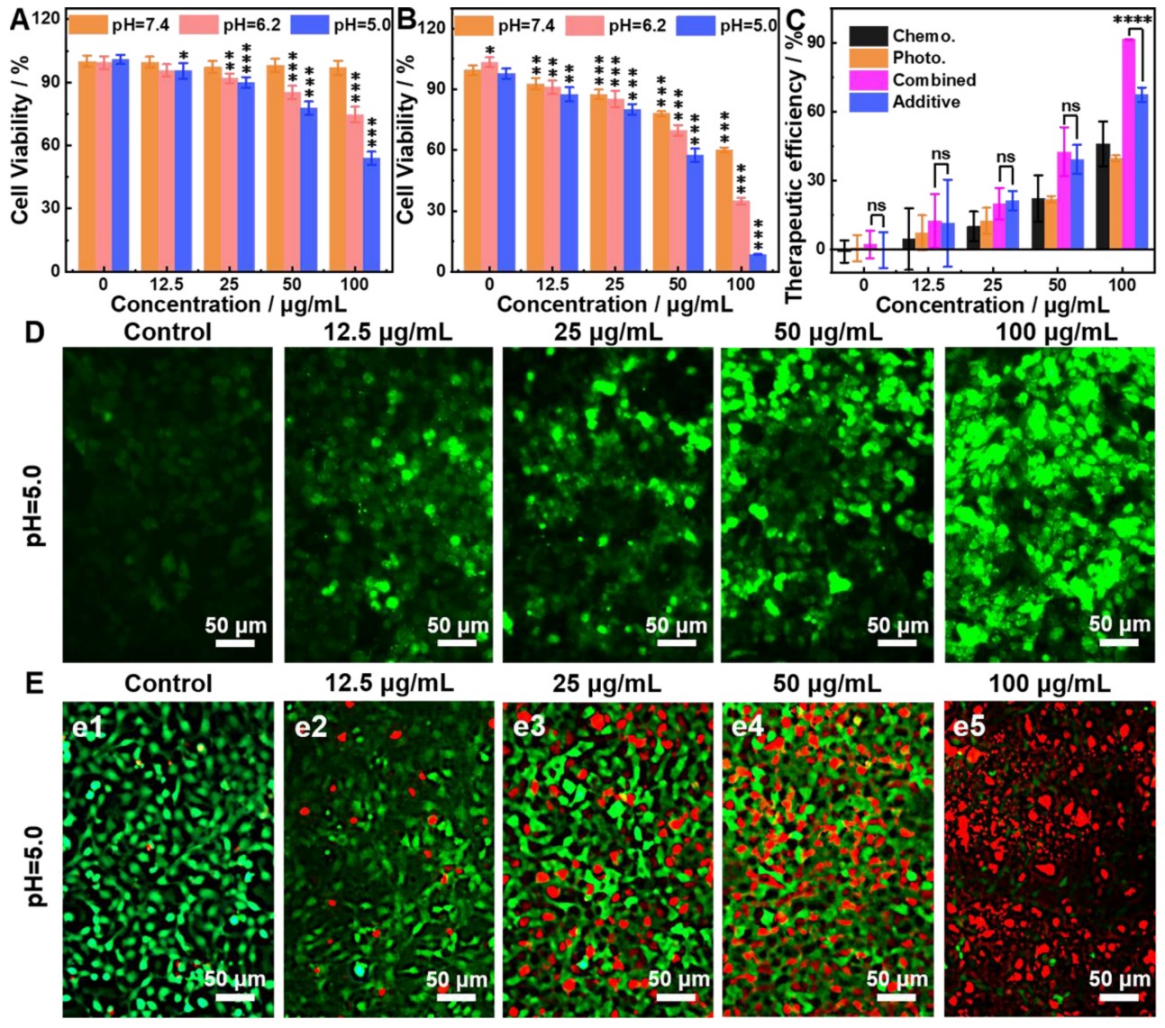
***In vitro* therapy.** (**A and B**) Relative cell viabilities of 4T1 cells treated by 9T-PUNNC at different concentrations and pH values without (A) and with (B) NIR-II irradiation. (**C**) Comparison of therapeutic efficacies of the 9T-PUNNC as a synergistic PTT and CDT agent with single PTT, CDT and additive treatment. (**D**) Confocal fluorescence images of DCFH-DA stained 4T1 cells after treated with the 9T-PUNNC for 20 min at pH = 5.0 with NIR-II irradiation. (**E**) Confocal fluorescence images of calcein-AM/PI double-stained 4T1 cells after treated with the 9T-PUNNC at pH = 5.0 with NIR-II irradiation. The scale bar is 50 µm. Statistical analysis was performed using the unpaired Student's t-test with *P < 0.05, ** P < 0.01 and *** P < 0.001 considered as statistically significant.

The CDT of 9T-PUNNC is implemented *in vitro*. More than 94% of cells survived after being treated by the 9T-PUNNC (100 μg mL^-1^) for 72 h at pH = 7.4 while the cell viability decrease intensely (~54%) at pH = 5.0 (Figure 6A). Importantly, the introduction of NIR-II irradiation further significantly reduced the viability of 4T1 cells at the same treatment conditions (Figure 6B). Notably, cell viability of 4T1 cells was assessed under different pH conditions, the results indicated that weakly acidic environment had no effect on cell survival (Supplementary Figure S17). Figure 6C shows a comparison of the therapeutic efficacies from the combined treatment with additive therapeutic efficacies of independent PTT and CDT. The therapeutic efficacy of the combined therapy was significantly higher than the additive therapeutic efficacy of PTT and CDT alone. Such synergistic effect should be attribute to the local temperature increase by NIR-II irradiated 9T-PUNNC, which not only promote the release of Ni^2+^ but also accelerate the Fenton-like reaction 52.

The reactive oxygen species (ROS) probe 2′,7′-dichlorofluorescin diacetate (DCFH-DA) was used to detect the generation of ·OH by the 9T-PUNNC in cancer cells 16, 53. 9T-PUNNC-treated 4T1 cells emitted stronger green fluorescence at an acid solution in comparison with a neutral solution (Figure 6D, Supplementary Figure S18). Notably, additional NIR-II irradiation accelerated the generation of ·OH at the same conditions (Figure 6D), resulting from the local temperature increase and the enhanced Fenton-like reaction (Supplementary Figure S19) 54. Accordingly, the confocal fluorescent images of 9T-PUNNC-treated 4T1 cells co-stained by calcein-AM and PI revealed more cell death at pH = 5.0 and NIR-II laser irradiation (Figure 6E) in comparison with no NIR-II irradiation (Supplementary Figure S20), indicating a strong synergistic effect of the combined chemodynamic-photothermal treatments.

**Figure 7:**
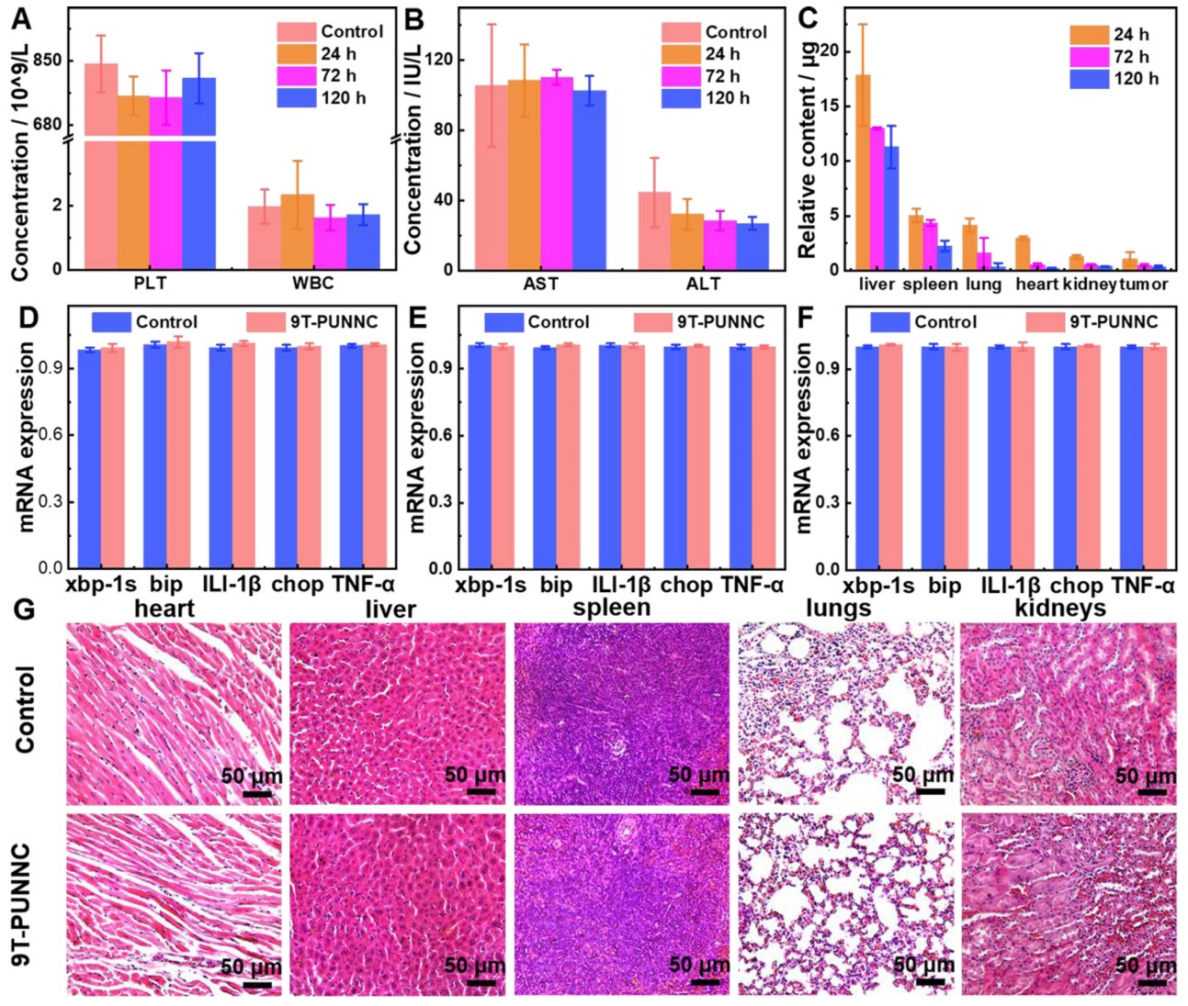
***In vivo* biocompatibility of the 9T-PUNNC.** (**A**) PLT and WBC counts of mice treated with PBS or the 9T-PUNNC for different times (n = 3). (**B**) Measurement of the levels of AST and ALT of mice treated with PBS or the 9T-PUNNC for different times (n = 3). (**C**) Biodistribution of the 9T-PUNNC in various organs and tumor tissues of nude mice after different exposure times. (**D-F**) RT-PCR analysis of the expression of ER stress response-related genes for main organs from mice treated with PBS or 9T-PUNNC for 120 h: (D) spleen, (E) lungs, and (F) kidneys, respectively. (**G**) Histological sections of main organs (heart, liver, spleen, lungs, and kidneys) obtained from PBS or 9T-PUNNC-treated mice. Scale bar, 50 µm. Statistical analysis was performed using the unpaired Student's t-test (P > 0.05).

*In vivo* biocompatibility of the 9T-PUNNC was further assessed. First, the circulating half-life of 9T-PUNNC in the blood flow was determined as 0.52 h based on a double-compartment pharmacokinetic model, (Supplementary Figure S21). Figure 7A-B, S22 and S24 then manifest the treatment effect at different times by blood routine and blood biochemical analysis of mice exposed to the 9T-PUNNC. No significant differences were observed in blood indicators such as platelet (PLT) counts, white blood cell (WBC) counts, and blood urea nitrogen (BUN) levels. In addition, the levels of aspartate aminotransferase (AST) in all groups remained normal. The biodistribution of the 9T-PUNNC was evaluated after the exposure of the 9T-PUNNC for 1, 3, and 5 days and quantifying the residual concentration of the 9T-PUNNC in various organs and tumor tissues (Figure 7C). The results showed that the 9T-PUNNC accumulated most in the liver but less in the kidneys.

Quantitative reverse transcription-polymerase chain reaction (RT-PCR) assay was employed to detect the changes in the transcription levels of genes related to endoplasmic reticulum (ER) stress response 55. The main organs of mice treated with the 9T-PUNNC and PBS respectively for 120 h were analyzed. Figure 7D-F and S23 manifest that the gene expression level of the 9T-PUNNC-treated mice in the spleen, lung, kidney, and liver was normal. The tissue biocompatibility of the 9T-PUNNC was further evaluated through histological analysis. As shown in Figure 7G and S24, the organs treated by the 9T-PUNNC and PBS have similar cell morphology, indicating an excellent biocompatibility of the 9T-PUNNC.

**Figure 8:**
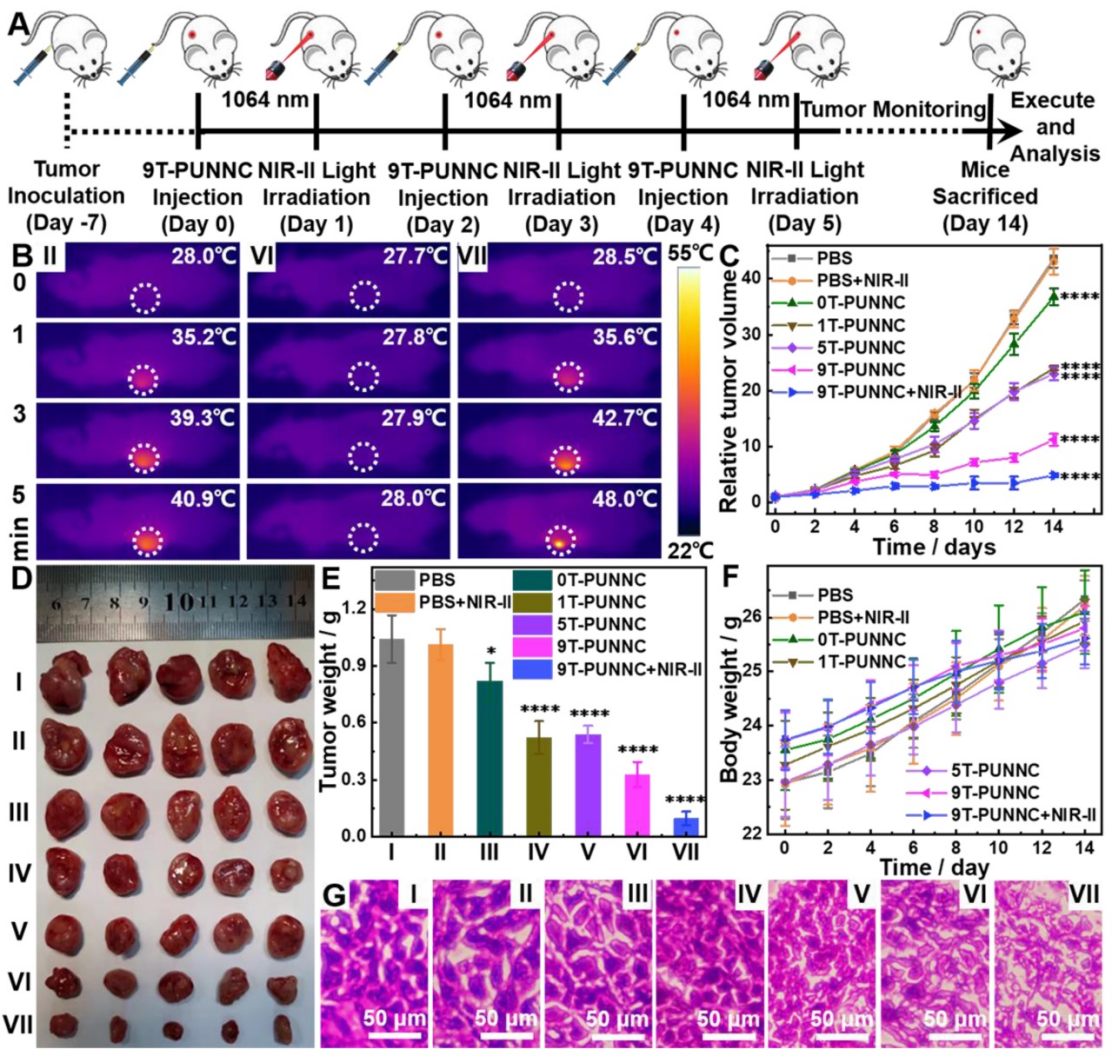
***In vivo* therapeutic effect of PUNNCs.** (**A**) *In vivo* treatment procedure of 9T-PUNNC. (**B**) Full-body thermographic images of 4T1-tumor-bearing mice treated with saline and 9T-PUNNC within 5-min NIR-II irradiation (1064 nm, 0.8 W cm^-2^) or not. Tumor growth curves (**C**), typical tumor photos (**D**), tumor weights (**E**), average mice body weights (**F**) and representative H&E staining of tumor sections (**G**) after receiving different treatments. I) PBS, II) PBS+NIR-II, III) 0T-PUNNC, IV) 1T-PUNNC, V) 5T-PUNNC, VI) 9T-PUNNC, VII) 9T-PUNNC+NIR-II. The scale bar is 50 µm. Statistical analysis was performed using the unpaired Student's t-test (*P < 0.05 and **** P < 0.0001), the rest data has no statistical significance (P > 0.05).

Given the practical cell killing ability and good biosafety profile, the PUNNC was used as a therapeutic agent for combined cancer therapy *in vivo* (Figure 8A). Mice treated with PBS remained expeditious tumor growth even after being irradiated with 1064 nm while a pronounced tumor suppression effect was observed in mice treated by the 9T-PUNNC, which should come from the NIR-II responsive photothermal conversion effect of 9T-PUNNC (Figure 8B). Furthermore, the PUNNCs synthesized under a higher magnetic field showed better tumor growth inhibitory effect, which comes from a single CDT (Figure 8C). The group treated by 9T-PUNNC plus NIR-II irradiation resulted in much more obvious inhibition of tumor growth due to the combined anticancer effect of PTT and CDT (Figure 8D).

The mass and volume of the tumors were consistent with the tumor growth curve (Figure 8E, Supplementary Figure S25). In addition, all mice in each group displayed an increase in body weight in a similar manner (Figure 8F). No significant weight loss was observed, confirming the good biocompatibility of the PUNNCs. The histological analysis showed that PBS and PBS+NIR-II-treated groups' tumor cells maintained normal cell morphology with obvious membrane and nuclear structures (Figure 8G). On the contrary, tumor cells were severely damaged and larger intercellular space exists in tumor tissues with the processing of PUNNCs synthesized under higher magnetic field. The group of 9T-PUNNC+NIR-II-treated were further destroyed, not only losing normal membrane and nuclear structures, but also causing permanent damage to the cells and inhibiting cell proliferation 26. Furthermore, Fluorescent staining of the frozen tumor tissues with O13 ROS fluorescent probe derived from mice treated with 9T-PUNNC showed more intracellular ROS production (Supplementary Figure S26). Together, these results indicate that the 9T-PUNNC holds excellent potential for combined cancer therapy.

## Conclusions

In summary, we have successfully synthesized a kind of PEGylation urchin-like nickel nanoclusters (PUNNCs) using a one-pot magneto-solvothermal strategy for synergistic photothermal-chemodynamic therapy. The external magnetic field controls the length of the needle protrusions on the PUNNCs' surface and decreases the crystallinity of the PUNNCs. The PEG coating of PUNNCs endows them with hydrophilic functional groups for good dispersity and stability in an aqueous solution and excellent biocompatibility *in vitro* and *in vivo*. The PUNNCs synthesized under a high magnetic field possess the increased NIR-II adsorption for enhanced photothermal conversion efficiency and accelerated acid-induced dissolution for improved ·OH generation. The PUNNCs enable a combined chemodynamic-photothermal treatment to provide superior therapeutic efficacy due to their highly synergistic effect.

## Experimental methods

### Materials

Nickel chloride hexahydrate (NiCl_2_·6H_2_O, ≥ 98%), hydrochloric acid (HCl, 36.0 ~ 38.0%) and sodium hydroxide (NaOH, ≥ 96%) were purchased from Sinopharm Chemical Reagent Co., Ltd. (Beijing, China). Hydrazine (N_2_H_4_·H_2_O, 80%), ethanol (C_2_H_6_O, ≥ 99.7%) and polyethylene glycol (HO(CH_2_CH_2_O)_n_H, 44.05 (MW)) were purchased from Titan Scientific Co., Ltd. (Shanghai, China). 3,3',5,5'-tetramethylbenzidine (TMB, ≥ 99.0%), acetic acid (CH_3_COOH, AR, 99.5%), sodium acetate trihydrate (CH_3_COONa·3H_2_O, GR, 99.5%), hydrogen peroxide (H_2_O_2_, AR, 30 wt.% in H_2_O), 5,5-dimethyl-1-pyrroline N-oxide (DMPO, 97%), Dimethyl sulfoxide (DMSO, AR, > 99.0%) were obtained from Aladdin Industrial Corporation (Shanghai, China). All chemical reagents were used as received without further purification, and ultra-pure water was used throughout all experiments.

### Synthesis of the 9T-PUNNCs

0.357 g of nickel chloride hexahydrate and 1.5 g PEG were dissolved in 15 mL ethanol. Then 2.5 mL of hydrazine hydrate containing sodium hydroxide (30 mg) was added dropwise into the mixed ethanol solution. After being stirred for 20 min, the mixed solution was transferred to an autoclave with a volume of 20 mL and heated at 80°C under an external magnetic field. After 20 h, the autoclave was naturally cooled to room temperature. The products were separated by a magnet (0.3 T), and washed with ethanol and deionized water three times. The collected black products were placed in an oven at 60°C for 6 h. Finally, the dried samples were defined as XT-PUNNCs, where X represents the used magnetic field strength (0, 1, 5, and 9).

### Sample characterization

The XRD patterns were characterized by using Japan Rigaku D/Max-γA X-ray diffractometer equipped with Cu-Kα radiation (λ = 1.54178 Å) over the 2θ range of 10°-80°. Field emission scanning electron microscopy (FE-SEM) images were acquired on a JEOL JSM-6700M scanning electron microscope at an accelerating voltage of 5 kV. Transmission electron microscopy (TEM) images were obtained on a Hitachi H-7650 transmission electron microscope at an accelerating voltage of 100 kV. High-resolution TEM (HRTEM) images were taken on a JEOL-2010 transmission electron microscope. The magnetic properties were measured by a superconducting quantum interference device (SQUID) magnetometer (Quantum Design MPMS) at room temperature. The UV-vis spectra were recorded with a RAYLEIGH UV-2601 at room temperature. The size distribution and surface charge were tested on a dynamic light scattering instrument (Nano-2s90, Malvern) at room temperature. The Electron paramagnetic resonance (ESR) experiments were performed using Bruker EMX plus 10/12 (equipped with Oxford ESR910 Liquid Helium cryostat). The Fourier transform infrared (FT-IR) spectra were recorded with a Nicolet Instrument Co. iS10 FT-IR spectrometer. The concentrations of nickel ions were measured using inductively coupled plasma-atomic emission spectrometry (ICP-AES, Atomscan Advantage). The thermal images were recorded by a thermal imaging camera (FLIR ETS320, Teledyne FLIR).

### Photothermal Properties

The PUNNC solutions at different concentrations (0, 25, 50, 100, and 200 μg mL^-1^) were exposed to a NIR-II laser (0.8 W cm^-2^) with a wavelength of 1064 nm for 5 min, and the temperature change of the solution was monitored by a thermocouple. The volume of the sample aqueous solution was set to 1 mL, and the distance from the laser source was 3 cm.

*SAR* is defined as the power consumption per unit mass of nickel (W g^-1^). The calculation formula is as follows 56:


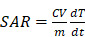

(3)

Where *C* represents the specific heat capacity of water (*C_water_* = 4185 J L^-1^ K^-1^), *V* is the volume of the solution, *m* is the mass of nickel present in the solution, and *dT/dt* is the initial linear slope of the temperature rise curve with time.

The photothermal conversion coefficient *η* of PUNNCs was calculated as follows 57:


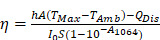

(4)

Where *h* is the heat transfer coefficient, *A* is the surface area of the cuvette, *T_Max_ - T_Amb_* represents the difference in temperature change, *Q_Dis_* is the heat dissipation of the light absorbed by the quartz cuvette itself after loading the solution, and *I_0_* is the power of the incident laser (W), *S* is the irradiation area (cm^2^), *A_1064_* is the UV absorption intensity of the PUNNCs at 1064 nm.

### Determination of hydroxyl radical production by the PUNNCs

The TMB by the PUNNCs in HAc-NaAc buffer produces a blue signal, and its prominent absorption peak is at 652 nm 58. In a typical test, the sample was added to 3.0 mL buffer solution (0.2 M HAc-NaAc buffer, pH = 5.0) in the order of a certain amount of the PUNNCs and 150 μL TMB (0.694 mM). The kinetic determination with TMB or H_2_O_2_ as the substrate was performed by adjusting the amount of TMB solution or H_2_O_2_ added. The maximum catalytic velocity and the Michaelis-Menten constant of the PUNNCs were obtained using the double reciprocal method (Lineweaver-Burke fitting).

### Free radical identification

The capillary tubes containing 20 µL control (distilled water containing DMPO) or sample solutions were inserted into the ESR cavity. The instrument settings were as follows: 1 G field modulation, 100 G scanning range and 20 mW microwave power for detection spin adducts using a spin trap. The DMPO was used to verify the generation of •OH during the degradation of H_2_O_2_ in the presence of the PUNNCs. The amount of •OH was quantitatively estimated by ESR signal intensity of the hydroxyl radical spin adduct (DMPO/•OH) using the peak-to-peak height of the second line of the ESR spectrum.

### Cell culture

The mouse breast cancer cell lines (4T1) were seeded into a 96-well plate and cultivated in RPMI-1640 media and Dulbecco's modified Eagle medium (DMEM) without L-glutamine (15-017-CVR; Corning, NY), respectively. The medium was supplemented with 10% (vol/vol) Fetal Bovine Serum (04-001-1A; Biological Industries, Israel), 2 mM GlutaMAX (35050-061; Gibco, Carlsbad, California) and 1% penicillin/streptomycin (SV30010; HyClone, Logan, UT). All cells were cultured at 37°C in a 5% CO_2_ incubator (Heracell 150i Thermo, USA).

### *In vitro* cell viability of the 9T-PUNNC

To evaluate cell viability of the 9T-PUNNC, 4T1 cells were seeded into a 96-well plate at a density of 0.3×10^4^ cells/well (80 μL per well) and incubated for 24 h. On the second day, 20 μL solution with different concentrations of the 9T-PUNNC (0, 12.5, 25, 50, and 100 μg mL^-1^) was added to the medium and further incubated for 72 h. The CellTiter-Glo luminescent assay was used to determine cell activity and a microplate reader (SpectraMax i3, Molecular Devices, Sunnyvale, CA) was used to detect the fluorescence intensity. To assess the sensitivity of 4T1 cells to acidic conditions, the same density of 4T1 cells was incubated with normal medium overnight. On the second day, the cells were cultured for indicated times under different pH conditions (5.0, 6.2, and 7.4), respectively. The cells were then harvested and the cell vaibility was determined by the CellTiter-Glo assay.

### *In vitro* PTT of the 9T-PUNNC

4T1 cells were seeded into a 96-well plate and incubated overnight in RPMI-1640 medium. On the second day, the 20 μL solutions with different concentrations of the 9T-PUNNC (12.5, 25, 50, and 100 μg mL^-1^) were added to 4T1 cells, and then being further incubated for 72 h at different pH values (5.0, 6.2, and 7.4). The cells treated with PBS served as a control. For photothermal treatment, the cells were irradiated with 0.8 W cm^-2^ NIR-II (1064 nm) laser for 5 min during the culture process. The CellTiter-Glo luminescent assay was used to assess cell viability. Each concentration in different groups was repeated six times.

### *In vitro* CDT of the 9T-PUNNC

First, 4T1 cells were seeded into a 96-well plate at a density of 0.3×10^4^ cell/well (80 μL per well), and cultured for 24 h at different pH values (5.0, 6.2 and 7.4). The 20 μL solutions with varying concentrations of the 9T-PUNNC (12.5, 25, 50 and 100 μg mL^-1^) were then added to the culture media, and the cells were continued to incubate for 72 h. The control group was only treated with PBS, and the concentration of each group was repeated six times. The Cell Titer-Glo assay was used to assess cell viability.

### Assessment of intracellular ROS levels

The ROS probe DCFH-DA was used to detect the ability of intracellular ROS generation 59. 4T1 cells were cultured in a six-well plate for 24 h. And then, 1 mL 9T-PUNNC solution (100 μg mL^-1^) was added into 4T1 cells and further cultured for 3 h. The DCFH-DA was added for 20 min and washed with PBS several times. Finally, the fluorescence image was obtained using a Leica TCS SP8X laser scanning microscope (Leica Microsystems GmbH, Germany) with λex=488 nm.

### Confocal laser scanning microscopic (CLSM) imaging of 4T1 cells treated with the 9T-PUNNC

4T1 cells were seeded onto glass coverslips in 6-well plates and incubated overnight in RPMI-1640 media at 37°C. The 20 μL solutions with different concentrations of the 9T-PUNNC (12.5, 25, 50, and 100 μg mL^-1^) were then added to the culture media and the cells were continued to incubate for 12 h. After 5 min of NIR-II irradiation, the cells were co-stained with calcein-AM (green, live cells) and PI (red, dead cells) for 15 minutes. The confocal fluorescent images were acquired using a Leica TCS SP8X laser scanning microscope (Leica Microsystems GmbH, Germany). Two different excitation wavelengths were used, including 490 and 545 nm.

### *In vivo* circulating half-life of 9T-PUNNC

Four-week-old female mice (n = 3) were intravenously injected with the 9T-PUNNC (200 μL, 1 mg mL^-1^). At 0.08, 0.25, 0.5, 1, 2, 4, 8, 24, and 48 h, 20 μL blood were obtained by nicking the tail vein. After acid treatment, the concentration of Ni^2+^ was measured by ICP. The *in vivo* circulating half-life of 9T-PUNNC in the blood was calculated by a double-compartment pharmacokinetic model 7.

### Hematological analysis, biodistribution, RT-qPCR assay and histopathological evaluation

Four-week-old female BALB/c (nu/nu) mice were purchased from Beijing Vital River Laboratory Animal Technology Co., Ltd. (Beijing, China). All animals were kept in the SPF facility of the Hefei Institutes of Physical Science Laboratory Animal Center. The research program involving animal care and use has been approved by the Ethics and Humanities Committee of the Hefei Institute of Physical Sciences (Chinese Academy of Sciences).

The female BALB/c mice were intravenously injected with the 9T-PUNNC (200 μL, dispersed in PBS) at a concentration of 1 mg mL^-1^. After 24, 72 and 120 h, quantitative analyses of blood including leukocytes, platelets, aspartate aminotransferase, alanine aminotransferase and blood urea nitrogen levels were performed (n = 3). PBS-treated mice were used as control (n = 3). 200 μL blood from each mouse with 0.15% (M/V) EDTA-K_2_·2H_2_O (EDTA double potassium salt, standard anticoagulant) was collected for a routine blood test. The whole blood samples were centrifuged (3500 rpm, 8 min, 4°C) to prepare a serum sample for the blood biochemical test.

After 24, 72, and 120 h, the main organs from 9T-PUNNC-treated mice (heart, liver, spleen, lung, and kidney) were harvested and placed in a 50 mL centrifuge tube, and then 1 mL of hydrochloric acid and 3 mL of nitric acid were added to centrifuge tube for acid dissolution. After 24 h incubation, all samples were centrifuged (12000 rpm, 20 min, and three times each). The supernatant (10 μL) was collected and further diluted 100 times. The content of nickel ions was tested by ICP, and the amount of the 9T-PUNNC deposited in each major organ was obtained.

After 120 h post-injection of the 9T-PUNNC, 20 mg of each tissue harvested from the mice was frozen using liquid nitrogen and grounded into a powder. The powder was then transferred to a microcentrifuge tube and processed with TRIzol (Thermo Scientific, Rockford, IL, USA) and Qiagen RNeasy Mini Kit (Qiagen, Hilden, Germany) to extract total RNA. cDNA was synthesized by Transcriptor First Strand cDNA Synthesis Kit (Roche, Mannheim, Germany). Quantitative real-time PCR was performed in triplicate using FastStart Essential DNA Green Master (Roche, Mannheim, Germany) on a Roche LightCycler 96 real-time PCR system.

After 120 h post-injection of 9T-PUNNC and PBS, all mice were sacrificed and the major organs (heart, liver, spleen, lung and kidney) were harvested. After being immersed in 4% paraformaldehyde for 24 h, the tissue was embedded in paraffin, sliced into 4 μm sections, and stained with hematoxylin and eosin. Microscopic images of tissues were acquired using a Nikon ECLIPSE TE2000-S microscope.

### *In vivo* PTT/CDT therapeutic efficacy of the 9T-PUNNC

Four-week-old female BALB/c (nu/nu) mice were used for *in vivo* assessment of the therapeutic effect of the 9T-PUNNC. Tumor-bearing mice were prepared by subcutaneous injection of 4T1 cells. The tumors were allowed to grow in the mice for 7 days. The tumor-bearing mice were randomly divided into 7 groups (n = 5 for each group). Two groups were injected with 200 μL PBS, two groups were injected with 9T-PUNNC (200 μL, 1 mg mL^-1^) and the others were injected with xT-PUNNC (x = 0, 1 and 5) three times as the experimental groups, respectively. After 24 h, a PBS-treated group and a 9T-PUNNC-treated group were selected to receive NIR-II (1064 nm, 0.8 W cm^-2^) irradiation for 5 min. The above operation was repeated every other day. The tumor sizes and the bodyweight of the mice were monitored every other day for 14 days. The length and width of the tumor were measured with a digital caliper, and the tumor volume was obtained by the formula V = ab^2^/2, where a is the length and b is the width of the tumor. After 14 days, all mice were sacrificed to get tumor tissue for photographing and HE analysis.

### In vivo ROS detection in tumor tissues

When the tumor volume reached about 100 mm^3^, mice (n = 3) were injected with 200 μL of PBS and 9T-PUNNC solution (1 mg mL^-1^), respectively. After 48 h treatment, tumors were harvested and prepared into 10 μm thick frozen sections. Tumor slice staining was conducted with O13 ROS fluorescent probe (BBoxiProbe®) and incubated at 37°C in the dark for 20 min. Subsequently, the staining solution was removed, and the fluorescence intensity was monitored under a fluorescence microscope after sealing with a coverslip 60.

### Statistical Analysis

All data are presented as mean ± standard deviation (n = 3 unless otherwise stated). The significance of the data was evaluated according to unpaired Student's two-sided t-test: *P < 0.05, **P < 0.01, ***P < 0.001, ****P < 0.0001, and NS (p > 0.05, no significant). All graphs were drawn with OriginLab Origin 2020.

## Supplementary Material

Supplementary figures and tables.
