# Supplementary material for: NIR-II responsive PEGylated nickel nanoclusters for photothermal enhanced chemodynamic synergistic oncotherapy
Source: Theranostics. 2022 May 1;12(8):3690–702. doi: 10.7150/thno.70841 (PMC9131281; doi:10.7150/thno.70841)
Supplement: Supplementary file 1 — Supplementary figures and tables. [file thnov12p3690s1.pdf]

Supplementary Data

Of

NIR-II responsive PEGylated nickel nanoclusters for photothermal enhanced chemodynamic synergistic oncotherapy

Yong Qian[a,b,1], Jiahui Zhang[a,b,e,1], Jinglu Zou[a,e,1], Xingyu Wang[a,b], Xiangfu Meng[a,b], Hongji Liu[a,b], Yefeng Lin[a,b], Qianwang Chen[a,b], Lei Sun*[,c], Wenchu Lin*[,a,e], and Hui Wang*[,a,b,d,e]

[a] High Magnetic Field Laboratory, Hefei Institutes of Physical Science, Chinese Academy of Sciences, Hefei 230031, Anhui, P. R. China

[b] University of Science and Technology of China, Hefei, 230026, Anhui, P. R. China

[c] Department of Stomatology, the Second Affiliated Hospital of Anhui Medical University, Hefei 230601, Anhui, P.R. China

[d] The Anhui Key Laboratory of Condensed Matter Physics at Extreme Conditions, Hefei Institutes of Physical Science, Chinese Academy of Sciences, Hefei 230031, Anhui, P. R. China

[e] Key Laboratory of High Magnetic Field and Ion Beam Physical Biology, Hefei Institutes of Physical Science, Chinese Academy of Sciences, Hefei 230031, Anhui, China

*Corresponding author: hw39@hmfl.ac.cn (H. Wang), wenchu@hmfl.ac.cn (W. Lin), sunlei_45@126.com (L. Sun)

[1] These authors contributed equally to this work.

Supporting text

Synthesis of the 9T-UNNC

The synthesis of 9T-UNNC was similar to that of 9T-PUNNC, the only difference was that no PEG was added during the synthesis. Specifically, 0.357 g of nickel chloride hexahydrate was dissolved in 15 mL ethanol. Then 2.5 mL of hydrazine hydrate containing sodium hydroxide (30 mg) was added dropwise into the mixed ethanol solution. After being stirred for 20 min, the mixed solution was transferred to an autoclave with a volume of 20 mL and heated at 80°C under an external magnetic field of 9 T. After 20 h, the autoclave was naturally cooled to room temperature. The products were separated by a magnet (0.3 T), and washed with ethanol and deionized water three times. The collected black products were placed in an oven at 60°C for 6 h.

Chemodynamic calculation method

The TMB concentration change rate and the H_2O_2 concentration change rate were plotted, respectively. It follows the Michaelis–Menten equation, known as [1, 2]:

$$v_0 = \frac{V_{max}[S]}{K_M+[S]} \qquad \text{(S1)}$$

Where v_0 is the initial speed of the reaction, V_{max} is the maximum velocity of the reaction, $[S]$ is the substrate concentration, and K_m is the Michaelis-Menten constant.

The Michaelis-Menten equation describes the relationship between substrate conversion rate and substrate concentration. Generally, the V_{max} reveals the catalytic activity of the enzyme.

Through a simple conversion, the Michaelis–Menten equation above can be converted to be [3]:

$$\frac{1}{v_0} = \frac{K_M}{V_{max}} \times \frac{1}{[S]} + \frac{1}{V_{max}} \qquad \text{(S2)}$$

According to the above formula, the K_m and V_{max} of the 9T-PUNNC catalytic reaction can be determined by the Lineweaver-Burk diagram. When H_2O_2 was used as the substrate, the calculated K_m and V_{max} of the 9T-PUNNC catalyzed reaction were 448.51 mM and 0.07676 μM s^{-1}, and the K_m and V_{max} values were 0.546 mM and 0.03665 μM s^{-1} with TMB being the substrate, respectively.

Table S1. Comparison of *PCE* of NIR-II responsive photothermal agent under1064 nm irradiation.

Name	Morphology	*PCE* (%)	References
MoO_3/WO_3	Layered	46.9%	[4]
polypyrrole	spherical	41.97%	[5]
Ag_2S@MSN-TGF	spherical	44.7%	[6]
Rh_3Se_8	nanodot	57.5%	[7]
SiNSs	nanosheet	21.4%	[8]
Bi@ZIF-8	cube	24.4%	[9]
Cu_2Se	hollow	50.89%	[10]
InSe	nanosheet	39.5%	[11]
TiN	irregular elliptical	22.8%	[12]
9T-PUNNC	PEGylated urchin-like	20.93%	This work

Table S2. Comparison of irradiation wavelength of Ni-based photothermal agent.

Material	Irradiation wavelength (nm)	Light power density (W cm^{-2})	*PCE* (%)	References
mNiO-Tb	785	2.0	48.88	[13]
NiS_2	808	0.75	~43.8	[14]
CuS-NiS_2	808	1.0	52.2%	[15]
mNiS_2 NSs	808	2.0	44.6	[16]
Ni_3C NCs	808	0.5	~16.9	[17]
NiPPD NPs	808	1.5	18.5	[18]
Nickel-bis	940	1.0	~12	[19]
Ni_8C_n	980	1.62	~19	[20]
PDMA-UCST-C	980	0.54	64.9	[21]
9T-PUNNC	1064	0.8	20.93%	This work

Table S3. Comparison of *PCE* and catalytic rate constant of PUNNCs synthesized under different magnetic field strengths.

Nanocatalyst	*PCE* (%)	Substrate type	V_{max} (μM s^{-1})	K_m (μM)
0T-PUNNC	8.68	H_2O_2	0.0300	2.39×10^5
		TMB	0.0236	660
1T-PUNNC	10.4	H_2O_2	0.0536	8.74×10^5
		TMB	0.0293	462
5T-PUNNC	13.8	H_2O_2	0.0640	6.70×10^5
		TMB	0.0313	568
9T-PUNNC	20.9	H_2O_2	0.0768	4.49×10^5
		TMB	0.0367	547
9T-PUNNC +37℃	-	H_2O_2	0.0823	4.27×10^5
		TMB	0.0397	308

Supplementary Figure

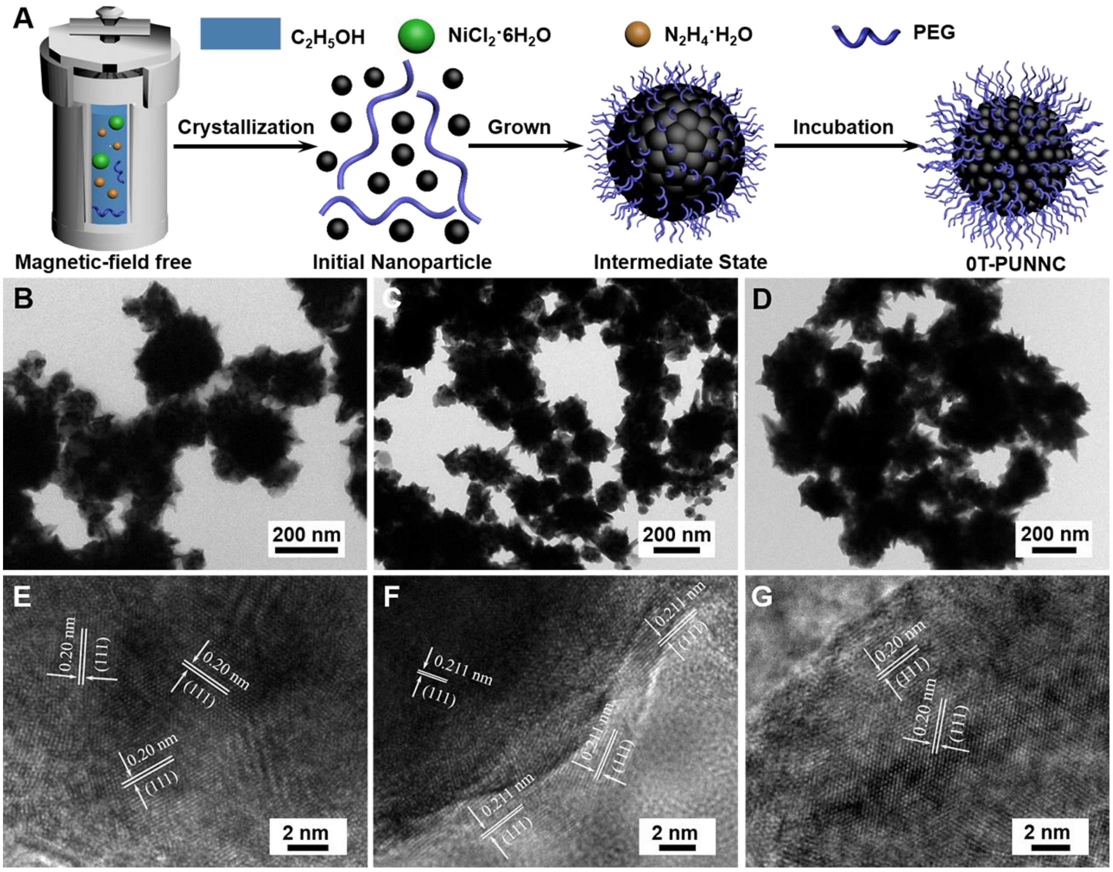

Figure S1. (A) Schematic illustration of the formation of the 0T-PUNNC. TEM and corresponding HRTEM images of the PUNNCs synthesized under different magnetic field conditions. (B) and (E) for 0T-PUNNC, (C) and (F) for 1T-PUNNC, (D) and (G) for 5T-PUNNC.

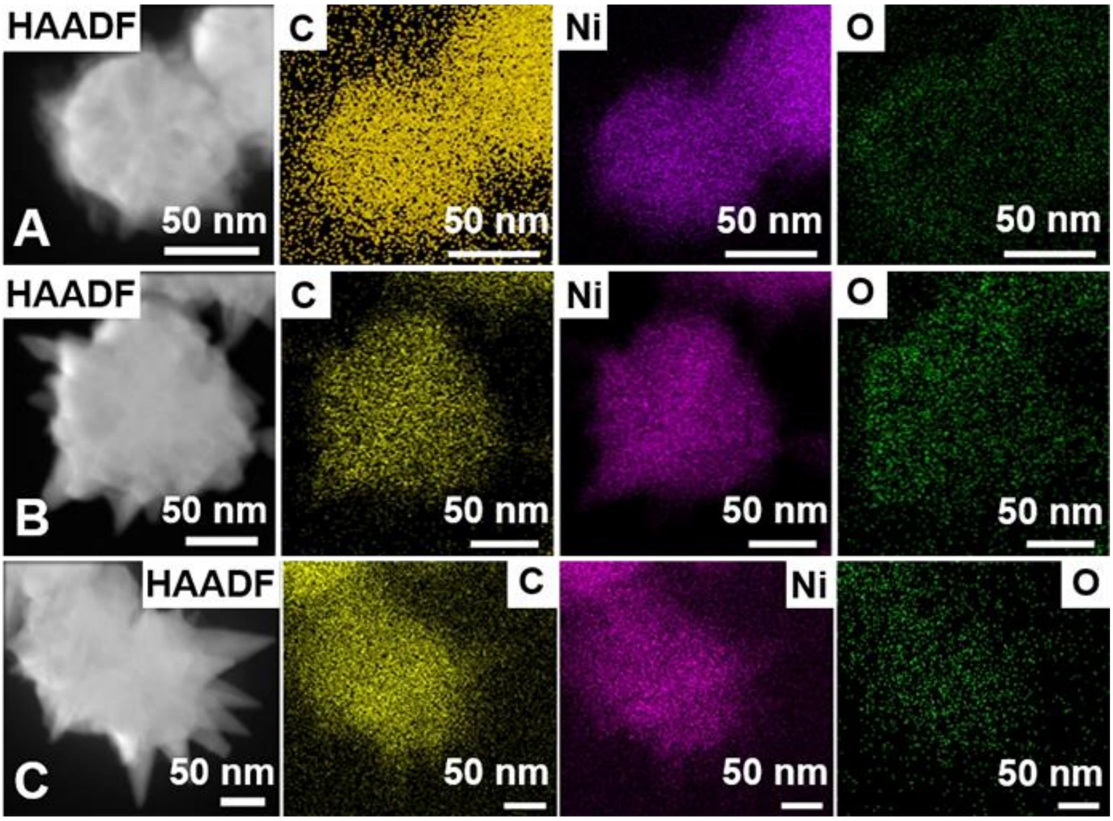

Figure S2. HAADF and corresponding element mappings (for C, O and Ni) of the PUNNCs. The scale bar is 50 nm: (A) 0 T, (B) 1 T and (C) 5 T.

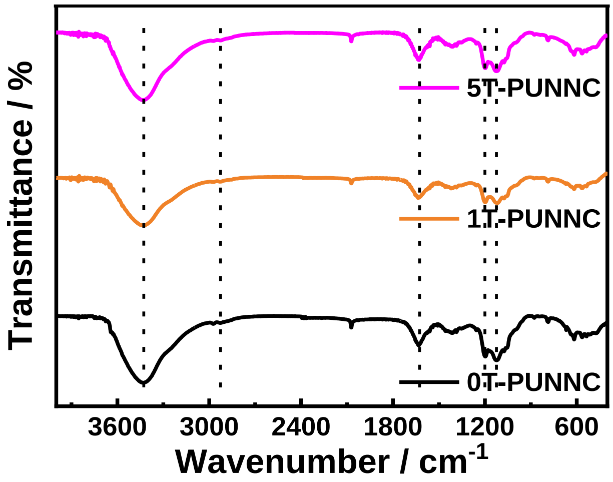

Figure S3. FT-IR spectra of as-synthesized PUNNCs synthesized under different magnetic field strengths.

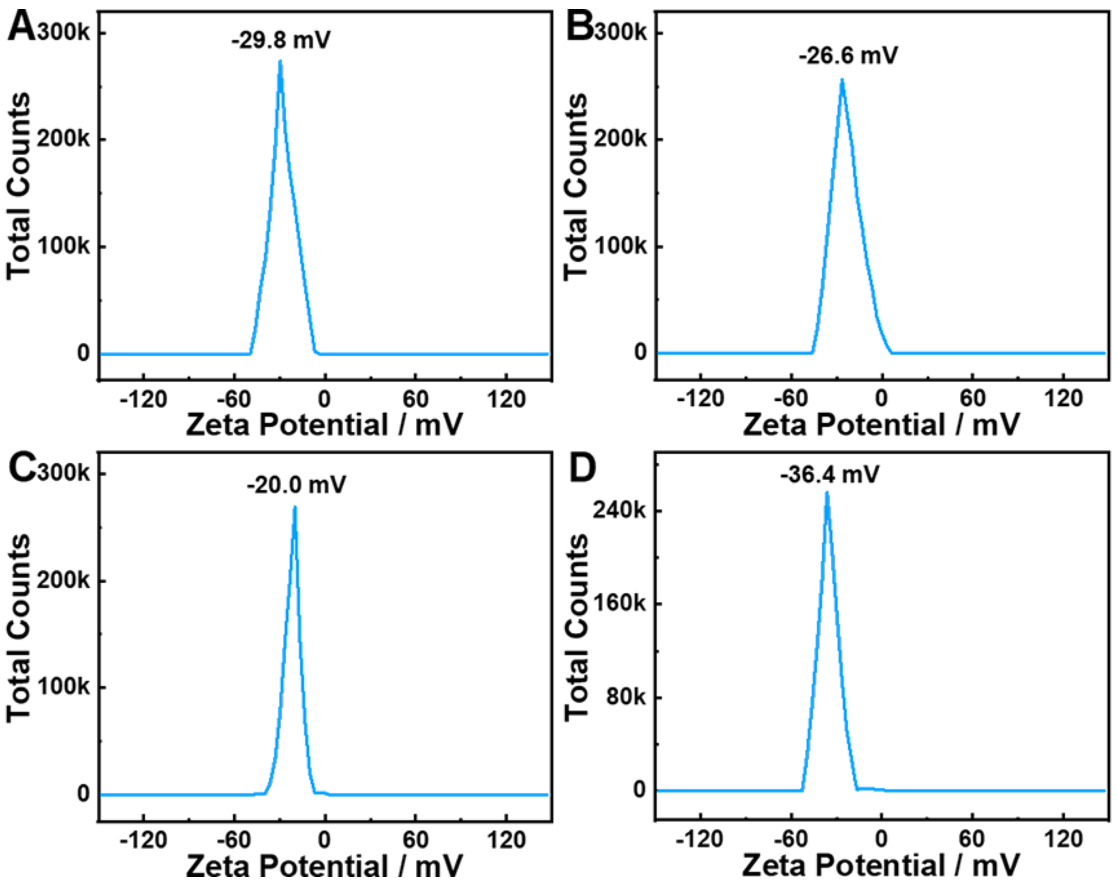

Figure S4. *Zeta* potential of the as-synthesized PUNNCs under different magnetic field strengths including (A) 0 T, (B) 1 T, (C) 5 T, (D) 9 T.

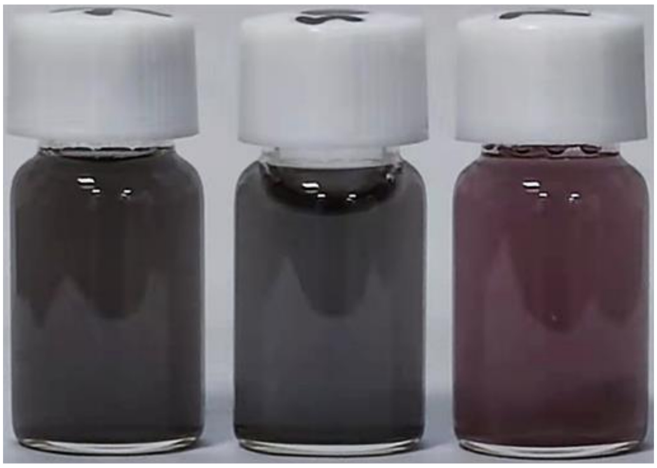

Figure S5. Photographs of the 9T-PUNNC dispersed in different solutions for 7 days. Left: distilled water. Middle: PBS. Right: RPMI-1640+FBS (10%).

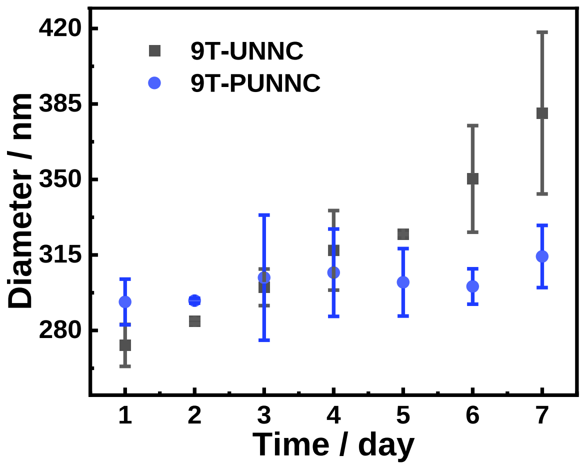

Figure S6. The hydrodynamic sizes of the 9T-PUNNC and 9T-UNNC within 7 days.

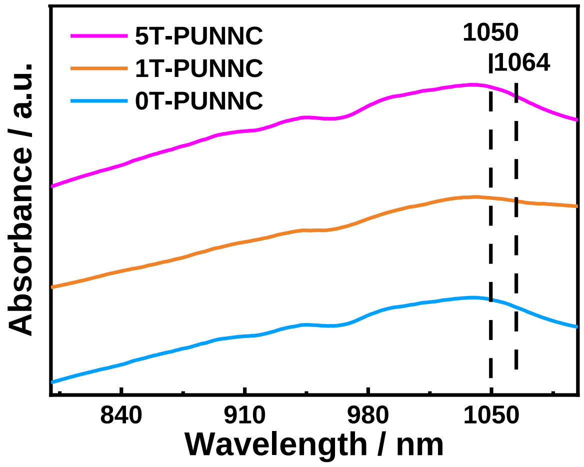

Figure S7. NIR absorptions of the as-synthesized PUNNCs under different magnetic field strengths including 0 T, 1 T and 5 T.

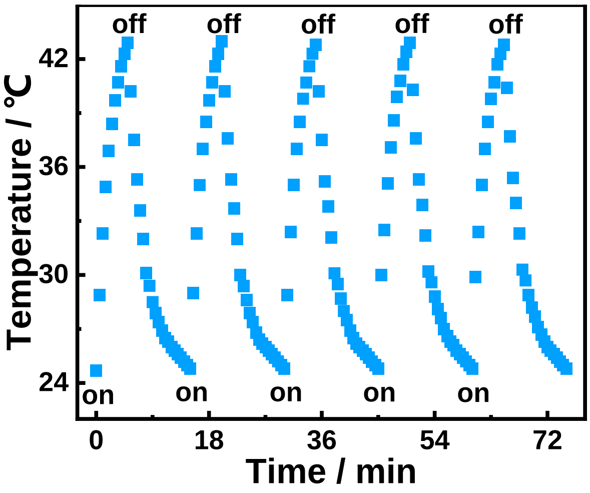

Figure S8. Heating curves of the 9T-PUNNC dispersed in distilled water for five cycles.

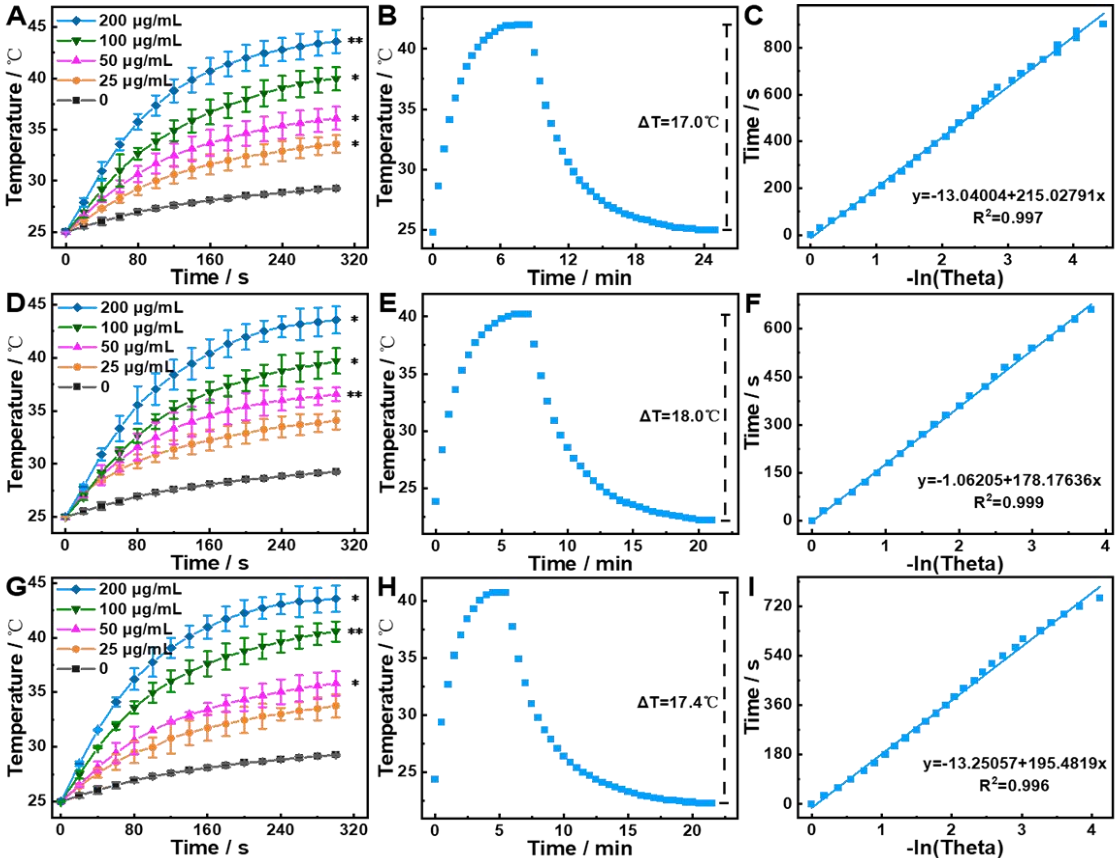

Figure S9. Photothermal characterizations of the PUNNCs. Temperature change of pure water and water containing different concentrations of the PUNNCs under NIR-II irradiation for 5 min ((A) 0 T, (D) 1 T and (G) 5 T). Temperature changes of

PUNNCs ((B) 0T, (E) 1T and (H) 5T) in aqueous (200 μg mL^{-1}) under NIR-II irradiation, the irradiation was turned off a period of time after initiation of irradiation (cooling period). (C), (F) and (I) Linear time data versus –*ln θ* obtained from the cooling period. The irradiation wavelength and power are 1064 nm and 0.8 W cm^{-2}, respectively.

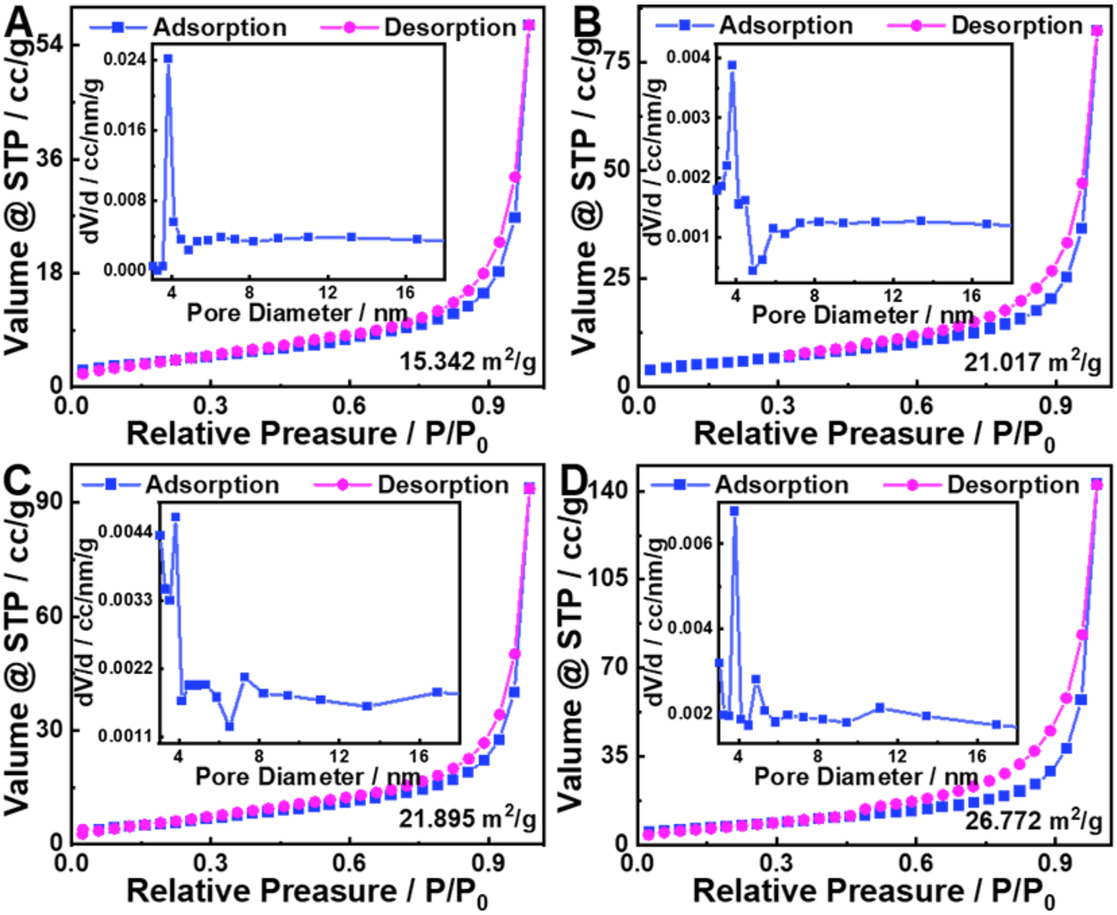

Figure S10. N_2 adsorption-desorption isotherm of the PUNNCs. The inset is the pore size distribution of the PUNNCs: (A) 0T-PUNNC, (B) 1T-PUNNC, (C) 5T-PUNNC and (D) 9T-PUNNC.

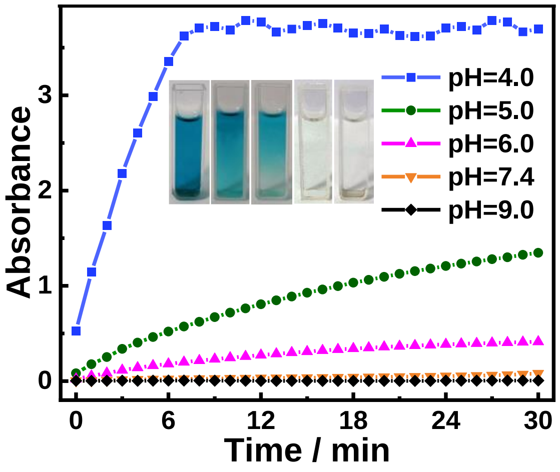

Figure S11. The changes of the absorbance intensity of TMB solution catalyzed by the 9T-PUNNC at different pH values.

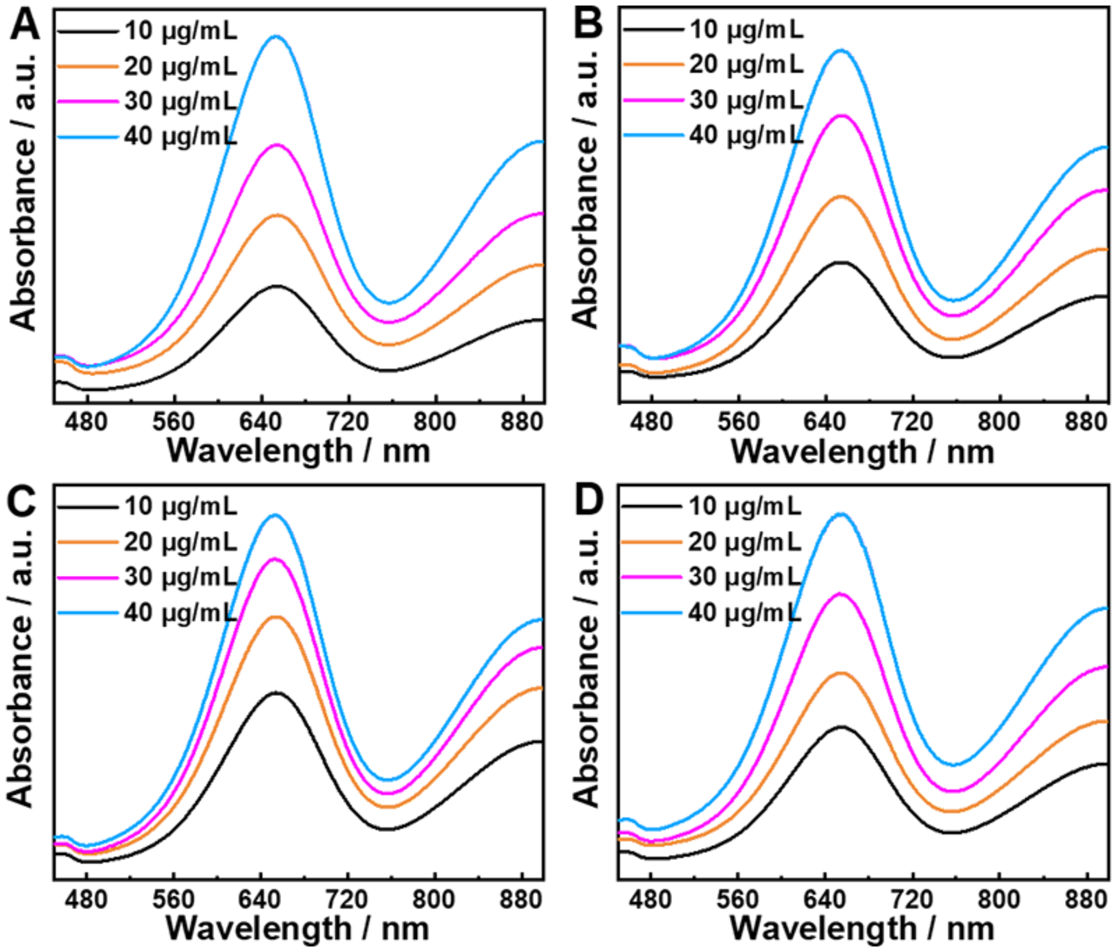

Figure S12. The steady-state catalytic kinetics of TMB and H_2O_2 were studied in a reaction system containing different concentrations of samples under acidic

conditions (pH=5.0) at room temperature: (A) 0T-PUNNC, (B) 1T-PUNNC, (C) 5T-PUNNC and (D) 9T-PUNNC.

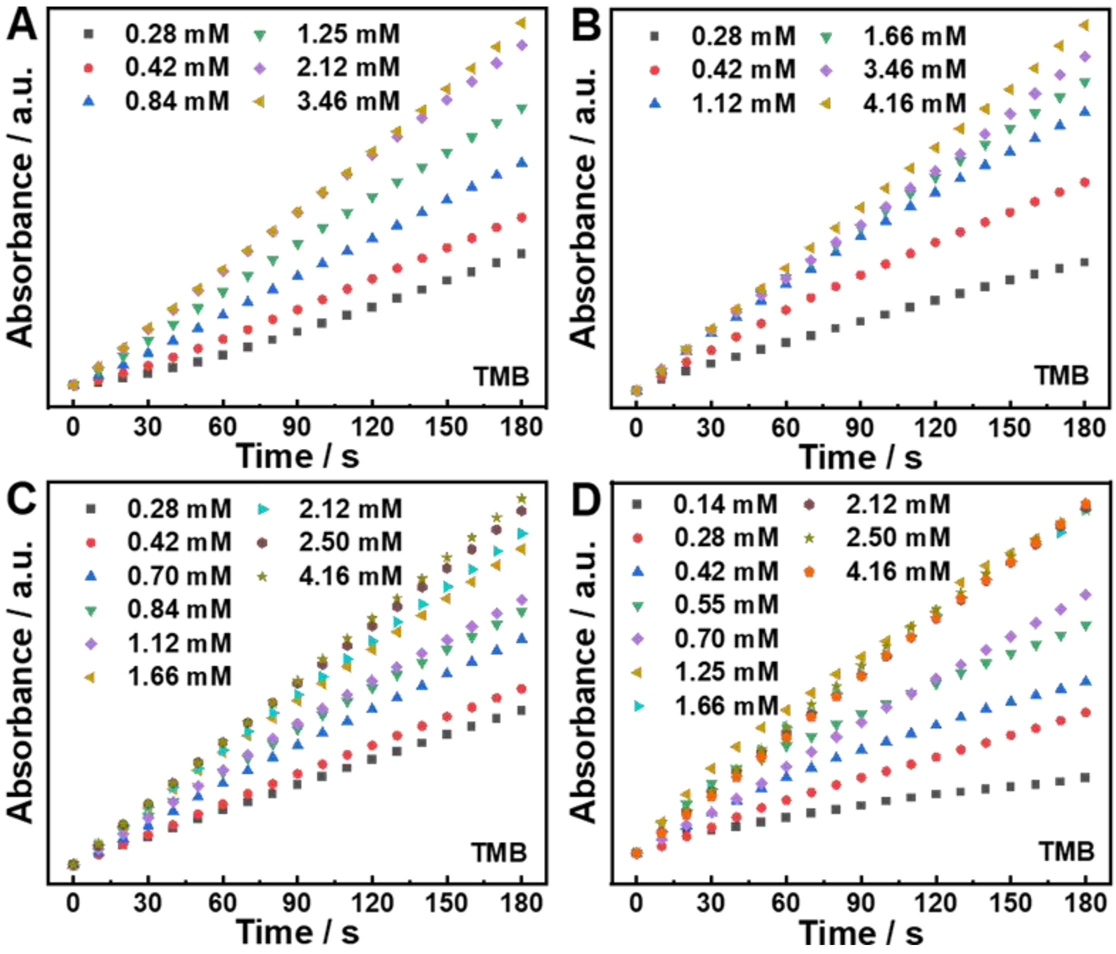

Figure S13. The initial linear portion of the reaction–time curves of TMB with different concentration were studied in a reaction system containing 6.67 μg mL^{-1} of samples under acidic conditions (pH = 5.0) at room temperature: (A) 0T-PUNNC, (B) 1T-PUNNC, (C) 5T-PUNNC and (D) 9T-PUNNC.

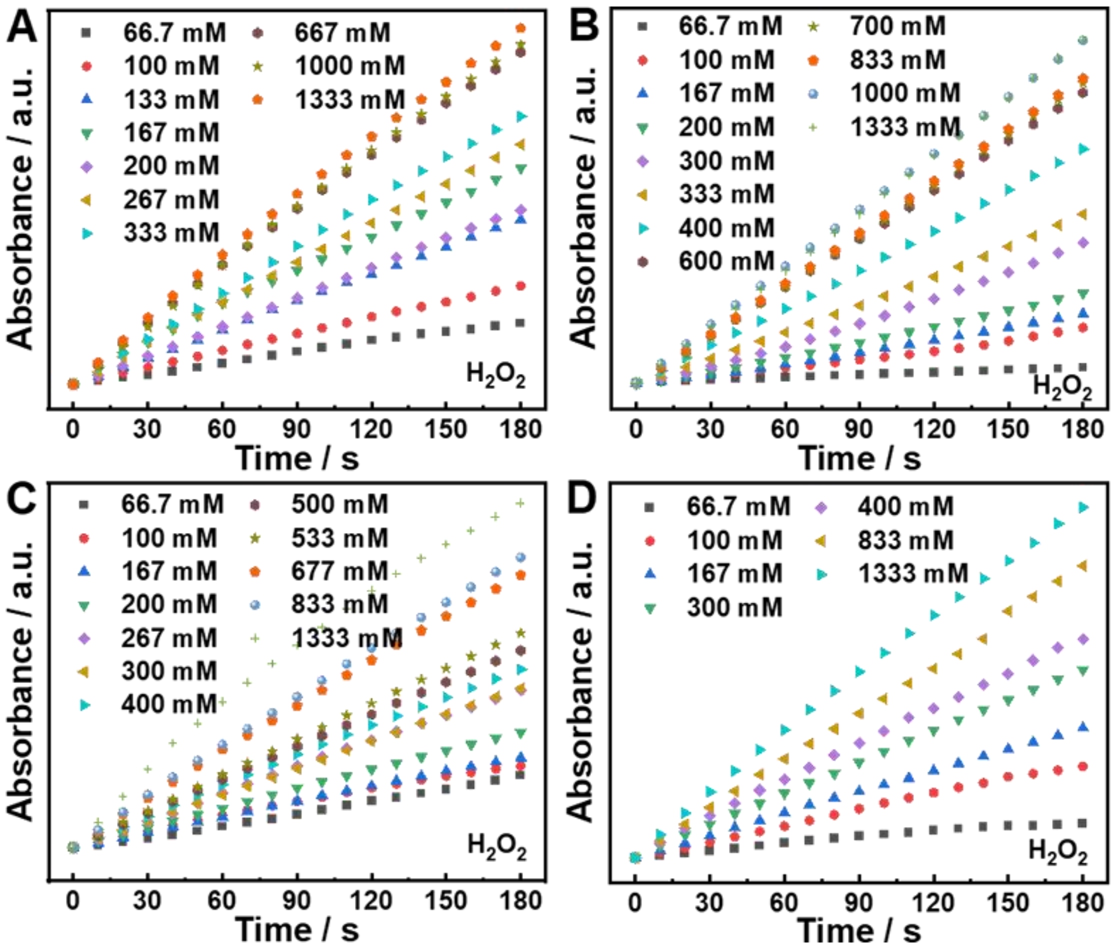

Figure S14. The initial linear portion of the reaction–time curves of H_2O_2 with different concentration were studied in a reaction system containing 6.67 μg mL^{-1} of samples under acidic conditions (pH = 5.0) at room temperature: (A) 0T-PUNNC, (B) 1T-PUNNC, (C) 5T-PUNNC and (D) 9T-PUNNC.

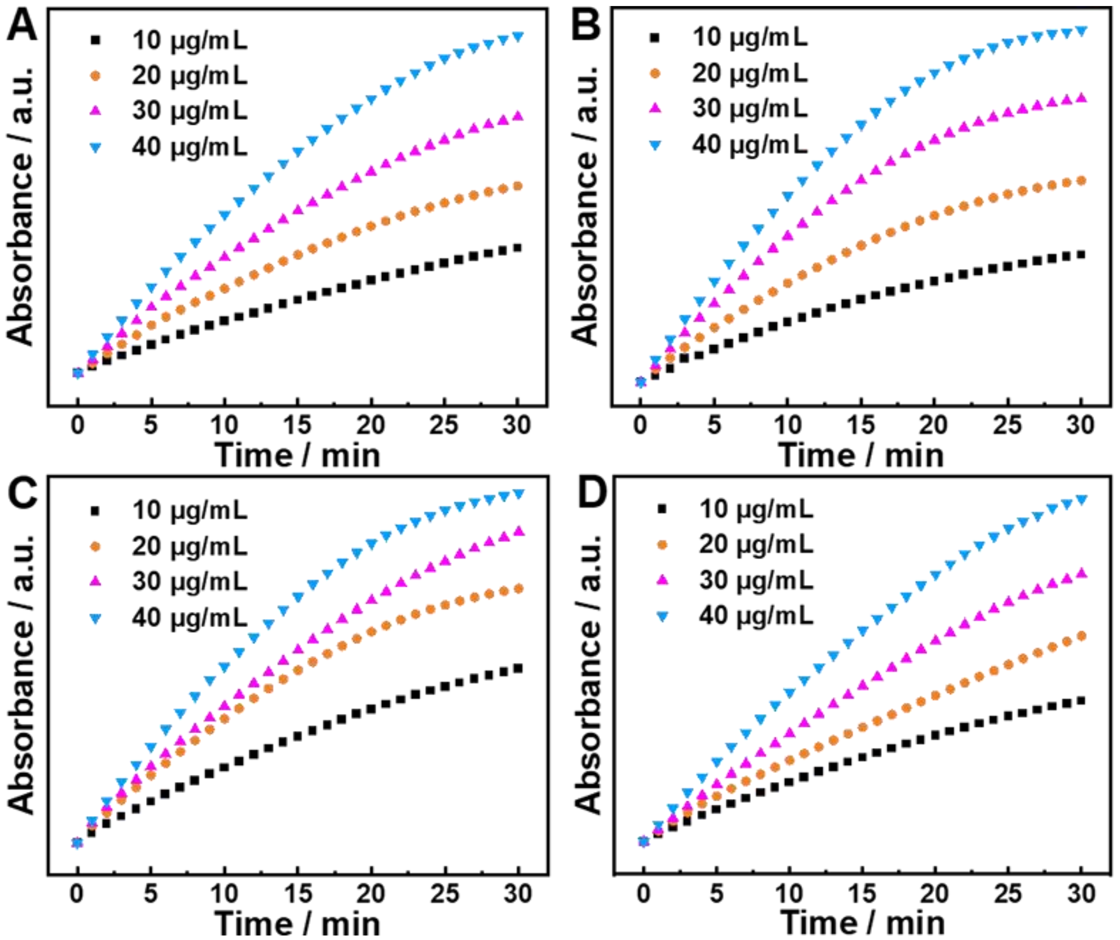

Figure S15. The changes of the absorbance intensity of TMB and H_2O_2 solution catalyzed by PUNNCs synthesized under different magnetic field strengths including (A) 0T, (B) 1T, (C) 5T and (D) 9T.

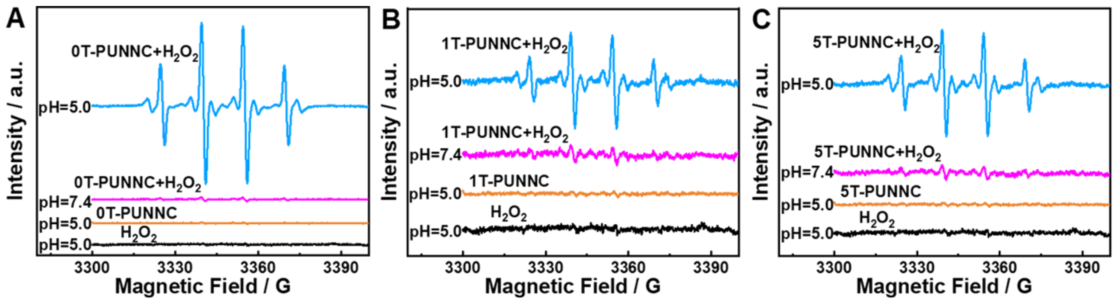

Figure S16. ESR signals of ·OH generation of the PUNNCs at acidic (pH = 5.0) and neutral condition (pH = 7.4) in the presence of DMPO. (A) 0T-PUNNC, (B) 1T-PUNNC and (C) 5T-PUNNC.

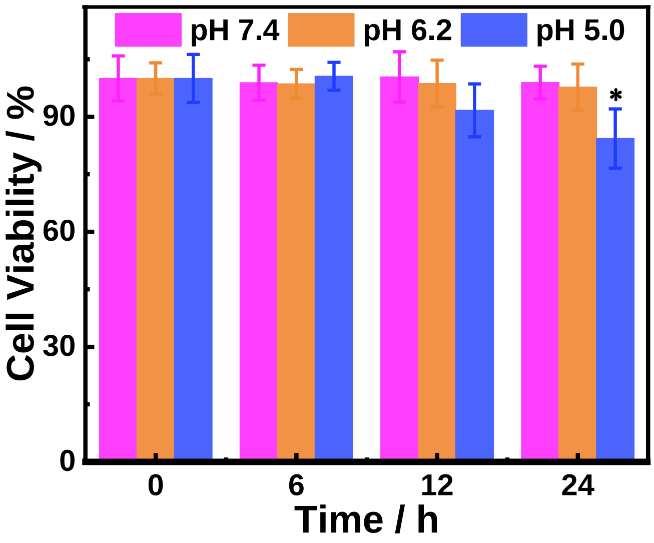

Figure S17. The cell viability of 4T1 cells under different pH conditions. Statistical analysis was performed using the unpaired Student's t-test with * P < 0.1 considered as statistically significant.

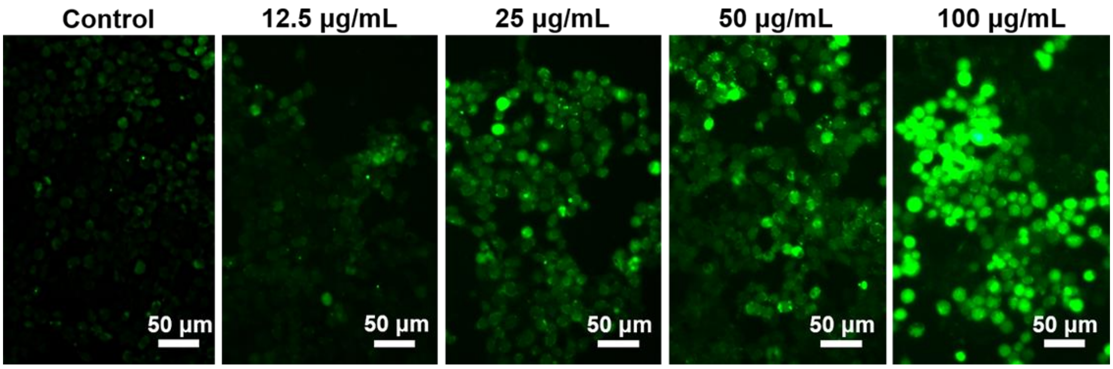

Figure S18. Representative confocal fluorescence images of DCFH-DA stained 4T1 cells after co-incubation with the 9T-PUNNC for 20 min at pH = 5.0. The scale bar is 50 μm.

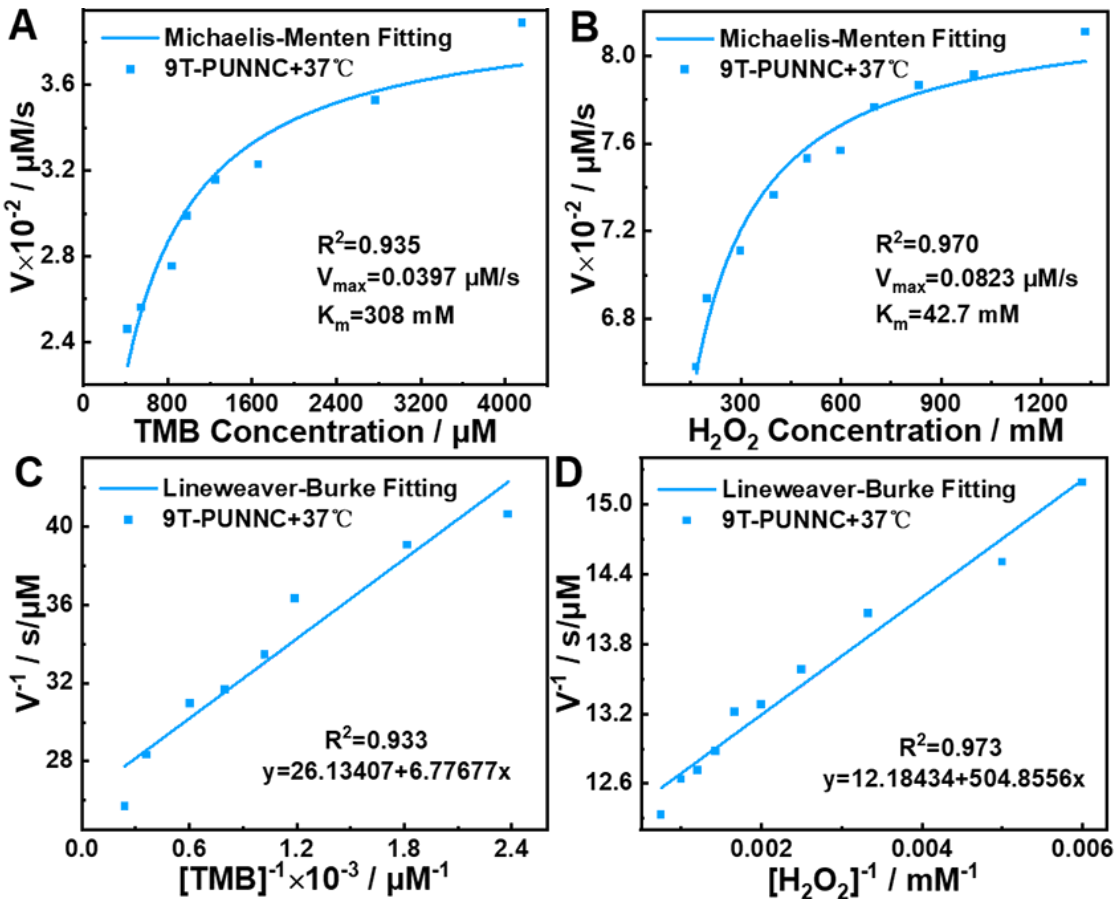

Figure S19. (A) The Michaelis-Menten fitting curves and (C) the Lineweaver-Burke fitting of initial ·OH generation velocities versus TMB concentrations under 50 mM H_2O_2 at 37°C. (B) The Michaelis-Menten fitting curves and (D) the Lineweaver-Burke fitting of initial ·OH generation velocities versus H_2O_2 concentrations under 2.12 mM TMB at 37°C.

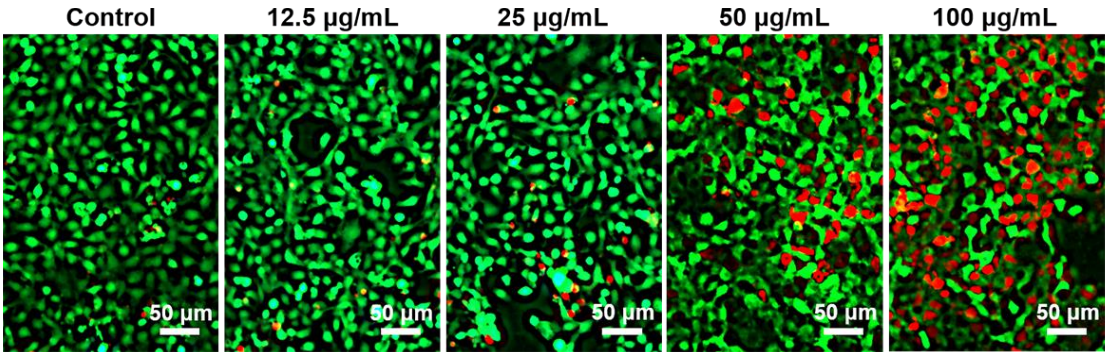

Figure S20. Representative confocal fluorescence images of 4T1 cells treated by the

9T-PUNNC at different concentrations at pH = 5.0 without NIR-II irradiation, and double co-stained with Calcein-AM/PI. The scale bar is 50 μm.

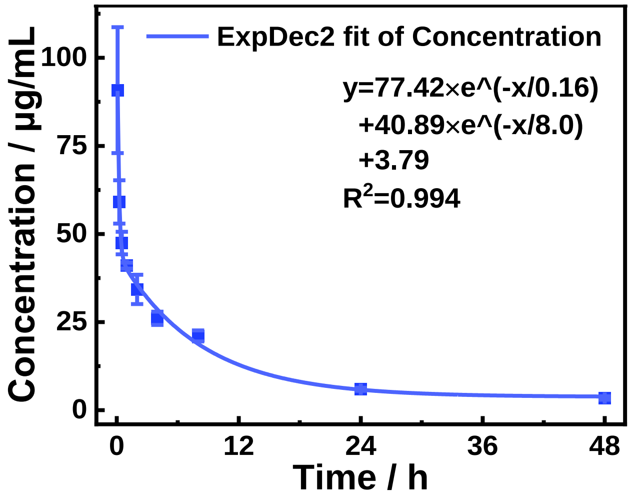

Figure S21. Blood circulation curve of mice treated by 9T-PUNNC for 48 h.

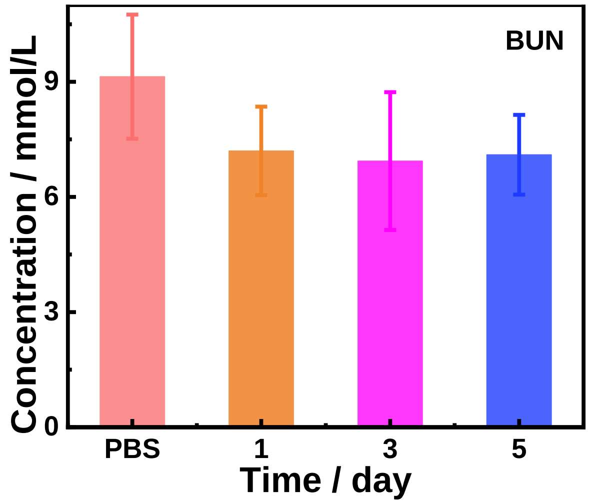

Figure S22. Blood urea nitrogen (BUN) levels of mice treated with PBS or 9T-PUNNC for different times (n = 3). Statistical analysis was performed using the unpaired Student's t-test ($P > 0.05$).

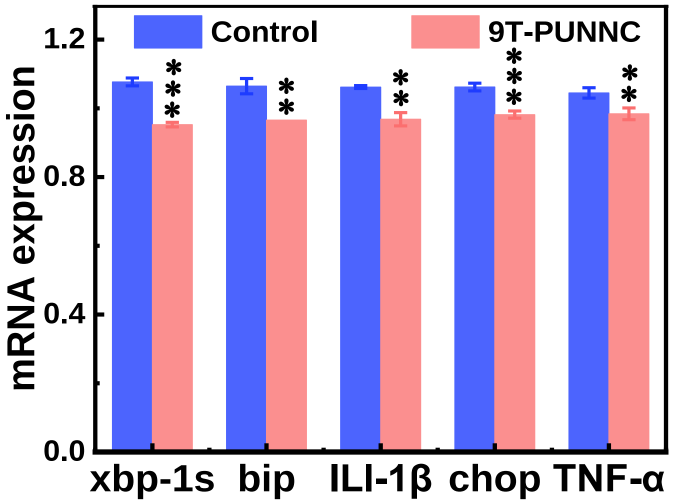

Figure S23. RT-qPCR analysis of expression of ER stress response-related genes for liver from mice treated with PBS or the 9T-PUNNC for 120 h. Statistical analysis was performed using the unpaired Student's *t*-test with ** $P < 0.01$ and *** $P < 0.001$ considered as statistically significant.

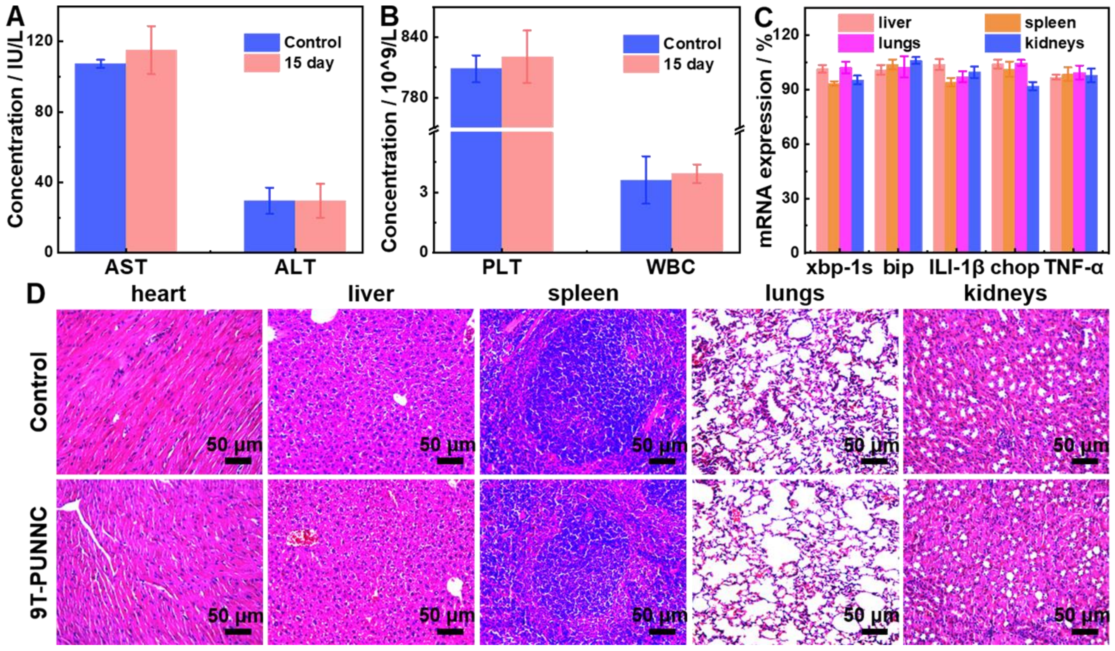

Figure 24. The long-term biosafety assessment of the 9T-PUNNC. (A) AST and ALT counts of mice treated with PBS or the 9T-PUNNC for 15 days. (B) Measurement of the levels of PLT and WBC of mice treated with PBS or the 9T-PUNNC for 15 days.

(C) RT-PCR analysis of the expression of ER stress response-related genes for main organs from mice treated with PBS or 9T-PUNNC for 15 days. (D) Histological sections of main organs (heart, liver, spleen, lungs, and kidneys) obtained from PBS or 9T-PUNNC-treated mice. The scale bar is 50 μm. Statistical analysis was performed using the unpaired Student's t-test (P > 0.05).

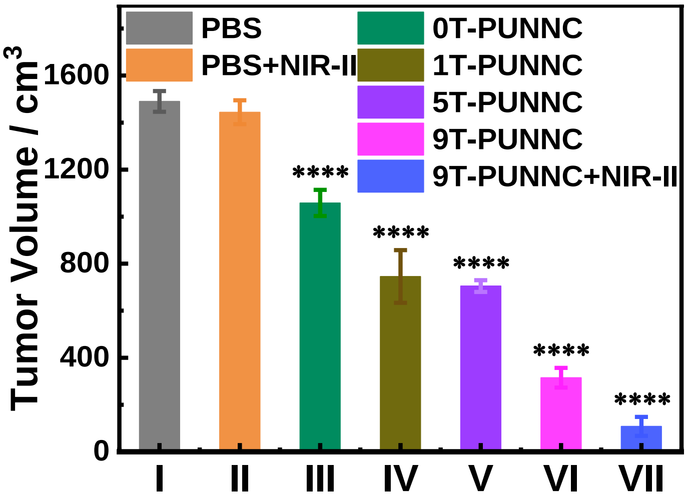

Figure S25. The average volumes of tumor collected from mice at the end of treatments (day 14). Statistical analysis was performed using the unpaired Student's *t*-test with **** $P < 0.0001$ considered as statistically significant.

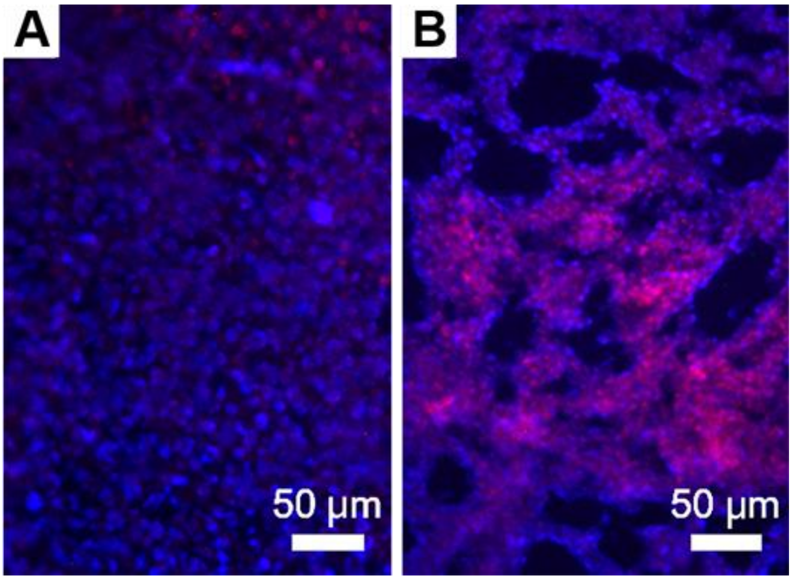

Figure S26. Representative confocal fluorescence images of O13 ROS fluorescent probe-stained tumor section after being treated with PBS (A) and 9T-PUNNC (B) for 48 h. The scale bar is 50 μm.

Supplementary Reference

[1] Jiang B, Duan DM, Gao LZ, Zhou MJ, Fan KL, Tang Y, et al. Standardized assays for determining the catalytic activity and kinetics of peroxidase-like nanozymes. Nat Protoc. 2018; 13: 1506-1520.

[2] Dong JL, Song LN, Yin JJ, He WW, Wu YH, Gu N, et al. Co_3O_4 nanoparticles with multi-enzyme activities and their application in immunohistochemical assay. ACS Appl Mater Interfaces. 2014; 6: 1959-1970.

[3] Yang BW, Chen Y, Shi JL. Nanocatalytic medicine. Adv Mater. 2019; 31: 1901778.

[4] Zhou Z, Wang XW, Zhang H, Huang HX, Sun LA, Ma L, et al. Activating layered metal oxide nanomaterials via structural engineering as biodegradable nanoagents for photothermal cancer therapy. Small. 2021; 17: 2007486.

[5] Zeng WW, Wu XX, Chen T, Sun SJ, Shi ZF, Liu J, et al. Renal-clearable ultrasmall polypyrrole nanoparticles with size-regulated property for second near-infrared light-mediated photothermal therapy. Adv Funct Mater. 2021; 31: 2008362.

[6] Li WT, Liu SK, Dong SM, Gai SL, Zhang FM, Dong YS, et al. A smart nanoplatform for synergistic starvation, hypoxia-active prodrug treatment and photothermal therapy mediated by near-infrared-II light. Chem Eng J. 2021; 405: 127027.

[7] Dong LL, Sun LN, Li WJ, Jiang YP, Zhan YY, Yu LD, et al. Degradable and excretable ultrasmall transition metal selenide nanodots for high-performance computed tomography bioimaging-guided photonic tumor nanomedicine in NIR-II biowindow. Adv Funct Mater. 2021; 31: 2008591.

[8] Cheng ZQ, Cui HD, Xiao QL, Huang H, Kang YH, Liu Q, et al. From octahedron crystals to 2D silicon nanosheets: Facet-selective cleavage and biophotonic applications. Small. 2020; 16: 2003594.

[9] Li JH, Zhu DM, Ma WJ, Yang Y, Wang GG, Wu XL, et al. Rapid synthesis of a Bi@ZIF-8 composite nanomaterial as a near-infrared-II (NIR-II) photothermal agent for the low-temperature photothermal therapy of hepatocellular carcinoma. Nanoscale. 2020; 12: 17064-17073.

[10] Wang XW, Zhong XY, Lei HL, Geng YH, Zhao Q, Gong F, et al. Hollow Cu_2Se nanozymes for tumor photothermal-catalytic therapy. Chem Mater. 2019; 31: 6174-6186.

[11] Huang C, Sun ZB, Cui HD, Pan T, Geng SY, Zhou WH, et al. InSe nanosheets for efficient NIR-II-responsive drug release. ACS Appl Mater Interfaces. 2019; 11: 27521-27528.

[12] Wang CM, Dai C, Hu ZQ, Li HQ, Yu LD, Lin H, et al. Photonic cancer nanomedicine using the near infrared-II biowindow enabled by biocompatible titanium nitride nanoplatforms. Nanoscale Horiz. 2019; 4: 415-425.

[13] Liu LD, Liu YX, Ma LY, Mao F, Jiang AQ, Liu DD, et al. Artemisinin-loaded mesoporous nanoplatform for pH-responsive radical generation synergistic tumor theranostics. ACS Appl Mater Interfaces. 2018; 10: 6155-6167.

[14] Wang XW, Fan LX, Cheng L, Sun YB, Wang XY, Zhong XY, et al. Biodegradable nickel disulfide nanozymes with GSH-depleting function for high-efficiency photothermal-catalytic antibacterial therapy. iScience. 2020; 23: 101281.

[15] Chen J, Zhang RJ, Tao C, Huang XF, Chen ZD, Li XP, et al. CuS–NiS_2 nanomaterials for MRI guided phototherapy of gastric carcinoma via triggering mitochondria-mediated apoptosis and MLKL/CAPG-mediated necroptosis. Nanotoxicology. 2020; 14: 774-787.

[16] He G, Ma Y, Zhou H, Sun SY, Wang XW, Qian HS, et al. Mesoporous NiS_2 nanospheres as a hydrophobic anticancer drug delivery vehicle for synergistic

photothermal–chemotherapy. J Mater Chem B. 2019; 7: 143-149.

[17] Zhou ZG, Wang J, Liu W, Yu C, Kong B, Sun YA, et al. PEGylated nickel carbide nanocrystals as efficient near-infrared laser induced photothermal therapy for treatment of cancer cells in vivo. Nanoscale. 2014; 6: 12591-12600.

[18] Yang RH, Li RD, Zhang L, Xu ZG, Kang YJ, Xue P. Facile synthesis of hollow mesoporous nickel sulfide nanoparticles for highly efficient combinatorial photothermal–chemotherapy of cancer. J Mater Chem B. 2020; 8: 7766-7776.

[19] Mebrouk K, Chotard F, Goff-Gaillard CL, Arlot-Bonnemains Y, Fourmigue M, Camerel F. Water-soluble nickel-bis(dithiolene) complexes as photothermal agents. Chem Commun. 2015; 51: 5268-5270.

[20] Mebrouk K, Debnath S, Fourmigue M, Camerel F. Photothermal control of the gelation properties of nickel bis(dithiolene) metallogelators under near-infrared irradiation. Langmuir. 2014; 30: 8592-8597.

[21] Augé A, Camerel F, Benoist A, Zhao Y. Near-infrared light-responsive UCST-nanogels using an efficient nickel-bis(dithiolene) photothermal crosslinker. Polym Chem. 2020; 11: 3863-3875.
